# Ecological and Health Risks of Polycyclic Aromatic Hydrocarbons in Particulate Matter in Chinese Cities

**DOI:** 10.1029/2024GH001126

**Published:** 2025-06-06

**Authors:** Yongfu Wu, Yuan Meng, Han Zhang, Lianglu Hao, Tao Zeng, Yan Shi, Yunhe Chen, Ni Qiao, Yibin Ren

**Affiliations:** ^1^ College of Agriculture and Biological Engineering Longdong University Qingyang China; ^2^ College of Resources and Environmental Science Gansu Agricultural University Lanzhou China; ^3^ Qingyang Xifeng District People's Hospital Qingyang China; ^4^ Qingyang Ecological Environment Bureau Qingyang China

**Keywords:** ecological risk, health risks, polycyclic aromatic hydrocarbons, particulate matter, Chinese cities

## Abstract

During the first two decades of the 21st century, the level of polycyclic aromatic hydrocarbon (PAH) pollution in urban atmospheric particulate matter (PM) in China significantly increased. By combining data from more than 6,695 individual samples covering 89 typical cities (population > 0.5 million people) across China, this study focuses on evaluating the health risks to urban residents and the ecological risks to the surrounding environment from PAHs in PM using the methods of the United States Environmental Protection Agency and sediment quality standards. The PAH contents and contamination levels in Central China (CC) were lower than those in South China (SC) and North China (NC). NC exhibited the most severe PAH pollution and greatest ecological risk, while CC had the highest population density and gross domestic product. The incremental lifetime cancer risk and hazard index values for people in NC were greater than those for people in CC and SC, and the health risk increased with increasing latitude. Based on ecological risk criteria and standard assessment methods, PAHs in PM in China pose a potential ecological risk, and the risk of harmful biological effects follows the order of NC > CC > SC. Given the significant risks of PAHs to people, animals and plants at both the national and global scales, under the guidance of the One Health concept of the World Health Organization, it is necessary to comprehensively manage PAHs in PM and reduce their threats to humans and ecosystems.

## Key Points

- The content of polycyclic aromatic hydrocarbons (PAHs) in particulate matter (PM) was highest in North China (NC) and lowest in South China (SC)
- The ecological risks of PAHs in urban PM in NC, Central China and SC were moderate or higher in most cities (>50%)
- Hazard index and lifetime incremental cancer risk values for urban residents were high in more than 73% and 3.4% of Chinese cities, respectively

## 1 Introduction

Following China's reform and opening‐up policy in 1978, all cities (population >0.5 million) have rapidly industrialized and urbanized, especially over the past two decades. Moreover, contamination from polycyclic aromatic hydrocarbons (PAHs, a class of aromatic compounds containing multiple carbon atoms) in urban areas has become increasingly severe. In terms of global PAH emissions, in 2004, 520 kilotons of PAHs were emitted into the atmosphere globally, with consumer product usage, wildfires, and fossil fuels as the primary sources (Umweltbundesamt, 2016); approximately 21.9% (114 kilotons) of these PAHs were emitted in China (Zhang and Tao, 2009). Because the PAH source determines the level of PAH pollution in particulate matter (PM), the PAH content in PM is closely related to the development status and energy consumption profile of different cities or regions (Chen, Lai, et al., 2022; Imai et al., 2023; Jin et al., 2018; Li et al., 2020; Lian et al., 2022; Ohura et al., 2018; Yuan et al., 2012).

Moreover, many studies have confirmed that PAHs absorbed in PM can be transported and remobilized in the atmosphere through volatilization (Ghanavati et al., 2019; Thang et al., 2019). PM, especially fine PM (PM_2.5_), is one of the most important carriers of PAHs (Ma et al., 2020; Zhu et al., 2019). In addition, PM can enter the human body via inhalation, ingestion, and dermal contact due to its long‐term suspension in the air (Mohammad et al., 2021), which can jeopardize human health (Razegheh et al., 2021). Thus, the health effects of PAHs deserve priority attention (Hong et al., 2021).

Since PAHs exhibit teratogenic, mutagenic, toxic, and/or carcinogenic properties (Zhou et al., 2022), their tendency to bind with PM poses a serious threat to human health if PAH concentrations reach toxic levels (Zhu et al., 2022). Epidemiological and toxicity studies have demonstrated that PAHs can adversely affect the immune system, causing eye irritation, inflammation, nausea, proinflammatory responses, and cell damage even at low exposure levels (Barbosa et al., 2023; Niu et al., 2017; Wang et al., 2024). Furthermore, the rapid industrialization and urbanization in China since 2000 have significantly increased PAH levels in cities (Chen, Zeng, et al., 2022; Lei et al., 2022; Yan et al., 2019). Therefore, in the context of the continuous increases in urban population (>901 million) and the proportion of the total population living in cities (63%), the health risks of PAHs in PM are becoming increasingly important (Zhao et al., 2019). It is essential to investigate the contents of PAHs in PM and to assess the associated ecological and human health risks.

By analyzing the pollution levels, ecological risks, and human health risks of PAHs in PM in Chinese cities, we can extract useful information for risk control and development strategies. Thus, this study aimed to (a) analyze the spatial distribution of PAHs in China, (b) examine the pollution levels and ecological risks of PAHs in three regions [North China (NC), Central China (CC), and South China (SC)], and (c) evaluate the health risks of PAHs for four age groups [children (<6 years), pupils (6–12 years), teenagers (12–18 years), and adults (>18 years)] in Chinese cities.

## 2 Data and Methods

### 2.1 Data Collection

**Figure 1:**
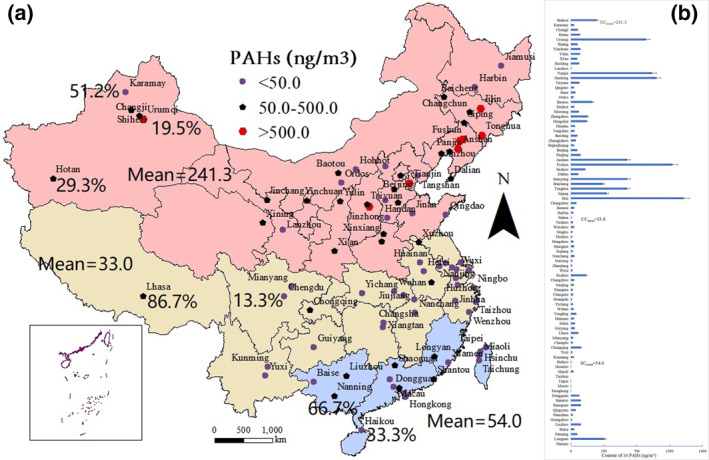
Spatial distribution and content of polycyclic aromatic hydrocarbon (PAH) in particulate matter (PM) of Chinese cities. (a) The purple stars denote PAH concentrations <50.0 ng/m^3^ (observed in 51.2%, 86.7%, and 66.7% of the cities in North China (NC), Central China (CC), and South China (SC), respectively). The black pentagons denote PAH concentrations of 50.0–500.0 ng/m^3^ (observed in 29.3%, 13.3%, and 33.3% of the cities in these three regions). The red hexagons denote PAH concentrations >500.0 ng/m^3^ (observed in 19.5%, 0.0%, and 0.0% of the cities in these three regions). The red, yellow, and blue areas indicate NC, CC, and SC, respectively, which had average PAH contents in PM of 241.3, 33.0, and 54.0 ng/m^3^. (b) Total PAH content in PM in each city in NC, CC, and SC.

A number of studies have reported the contents of PAHs in PM in cities. These studies were published from 2000 to 2019 and include analyses of PAH contamination in response to rapid socioeconomic development in China, considering population increases, growing energy demand, and increases in industrial production. Publications in the scientific literature were selected following the eligibility criteria described below. Original studies published in English databases (e.g., Elsevier, Springer, and Wiley) and excellent works published in Chinese databases (e.g., CNKI, CQVIP, and Wanfang Data) were included. We searched for papers and books with the keywords “polycyclic aromatic hydrocarbon in the PM,” “PAH in the PM,” “polycyclic aromatic hydrocarbon in PM,” “PAH in PM”, and “City, China.” Thus, we retrieved almost all the relevant reports about PAHs in PM in Chinese cities. After removing duplicate or incomplete data, we collected data on the contents of 16 PAHs in PM in 89 cities in 34 provinces of China (Table S1 in Supporting Information S1). Based on their meteorological and geographical conditions and latitude, these cities were divided into three regions: NC, including 16 provincial administrative regions: Heilongjiang, Jilin, Liaoning, Anhui, Beijing, Hebei, Henan, Inner Mongolia, Shandong, Shanxi, Tianjin, Gansu, Shaanxi, Ningxia, Qinghai, and Xinjiang; CC, including 11 provincial administrative regions: Hubei, Hunan, Jiangsu, Jiangxi, Shanghai, Zhejiang, Yunnan, Chongqing, Sichuan, Xizang, and Guizhou; and SC, including 7 provincial administrative regions: Fujian, Guangxi, Guangdong, Hongkong, Macao, Taiwan, and Hainan (Figure 1; Table S1 in Supporting Information S1).

### 2.2 Quality Assurance/Control

The quality assurance and control methods applied in the literature search were as follows. The most relevant and recent literature concerning PAH pollution in PM originating from Chinese cities (population >0.5 million people; CPGPRC, 2014) was considered in this review. PM samples were extracted with *n*‐hexane, dichloromethane, acetone, or a mixture thereof. For sample analysis, the average values of blank samples, duplicate samples and internal standards were used to ensure the reliability of the experimental data. The total PAH content was determined by gas chromatography‒mass spectrometry (GC–MS). Chromatographic conditions: 30 m × 250 μm, 0.25 μm Agilent DB‐5MS capillary column; carrier gas nitrogen flow rate, 1.0 mL/min; splitless injection; injection volume, 1 μL; heating program: initial column temperature 80°C, hold for 1 min, increase to 150°C (rate 25°C/min), increase to 200°C (rate 3°C/min), hold for 3 min, increase to 280°C (rate 8°C/min) and hold for 13 min. MS conditions: Electron bombardment source (electron energy, 70 eV); ion source temperature, 250°C; transmission line temperature, 280°C; solvent delay time, 3.5 min; monitoring mode, full‐scan mode with multiple reaction monitoring. GC conditions: column, SPB‐608 (5% phenyl/95% dimethyl polysiloxane), 30 m × 0.25 mm I.D., 0.25 μm (24103‐U); heating program, 70°C for 0.2 min, 25°C/min ramp to 265°C, 5°C/min ramp to 300°C, hold for 12 min; injection temperature, 250°C; flame ionization detector, 300°C; carrier gas, helium, 1.3 mL/min, constant flow; 2.0 μL pulsed splitless injection (30 psi until 0.2 min); 4 mm I.D. single taper liner; PAH standard sample, 1 μg/mL in methylene chloride. High‐performance liquid‒mass spectrometry (HPLC–MS) was performed using a triple quadrupole mass spectrometer QTRAP6500 (ABSciex) and a Nexera X2 HPLC system (Shimadzu) coupled with a PAL HTC‐xt (CTC Analytics) autosampler. The mass range of the instrument was set at 5 to 2,000 m/z. Spectra were recorded in positive ion mode as [M+H]^+^ ions for the detection of PAHs. The ion spray voltage was set at 5.5 kV, the cone voltage was 30 V, and the source block temperature was 100°C. The curtain gas pressure was set at 20 psi, the collision gas pressure was 9 psi, ion source gas pressures 1 and 2 were set at 50 psi, the decluttering potential was 100 V, the entrance potential was 10 V, the collision cell exit potential was 12 V, and the collision energy was 35 eV. Lipid samples (10 μL each) dissolved in acetonitrile were injected using the autosampler, and molecules were separated using a Bio C18 column (1.9 μm, 2.1 × 150 mm) at 60°C. The LC system was operated at a flow rate of 100 μL/min with the following gradient: 90% mobile phase A (methanol/acetonitrile/deionized water = 18/18/4 containing 5 mM ammonium acetate) and 10% mobile phase B (2‐propanol containing 5 mM ammonium acetate), hold for 2 min, linear increase to 82% mobile phase B over 17 min, and hold at 85% mobile phase B for 4 min. The column was re‐equilibrated to 10% mobile phase B for 10 min before the next injection. HPLC gradient elution procedure: 80% methanol +20% water, hold for 20 min; increase to 95% methanol +5% water at a rate of 1.2% methanol/min until peak completion.

Mobile phase flow rate, 1.0 ml/min; UV detector wavelengths, 254, 220, and 295 nm; fluorescence detector: excitation wavelength *λ*_ex_ = 280 nm and emission wavelength *λ*_em_ = 340 nm, after 20 min *λ*_ex_ = 300 nm and *λ*_em_ = 400, 430, and 500 nm (Ohura et al., 2019; Zhe et al., 2019; HJ 646‐2013, 2013; HJ 647‐2013, 2013). Moreover, the mass‐labeled recovery material, the range of PAH recoveries during extraction, and the coeluting compounds were consistent with the standards of the Ministry of Ecological and Environmental Protection of China (HJ 742‐2015, 2015; HJ 1010‐2018, 2018). The detection methods were suitable for PAH detection; there were no significant differences in the PAH detection results, and all analyses were performed in accordance with national standards.

These studies mainly addressed PAH pollution, which has been well studied in recent years, with a focus on the 16 PAHs designated by the United States Environmental Protection Agency (US EPA) as priority control pollutants (Keshavarzi et al., 2018). Data were collected from at least one city in each province to reflect the pollution level and risk of PAHs in urban PM nationwide. We uniformly curated and edited the retrieved PAH data in accordance with China's national standards (GB/T 8170‐2008, 2008). Moreover, in the statistical analysis of data from previous studies, we used the lower limit of the 95% confidence interval to ensure scientific rigor and accuracy. Although the levels of PAHs in inhalable particulate matter (PM_10_) can comprehensively reflect the negative effects of PAHs on human health and ecosystems, detailed data on PAHs in PM_10_ were very limited. Therefore, most of the data we selected represent the content of PAHs in PM_2.5_. Only in a few cities where PM_2.5_ data were lacking did we use data for PAHs in PM_10_. These PM_10_ data were merely supplementary and were included to improve the statistical robustness and results without significantly altering the trends in pollution levels, health, and ecological risks in NC, CC, and SC. Tables S1–S3 in Supporting Information S1 detail the concentrations, toxic equivalent quantities, concentration ranges and mean values of ∑_16_PAHs and 16 individual PAHs.

### 2.3 Methods

#### 2.3.1 Ecological Risk

The ecological risk can reflect the degree of harm caused by PAHs in urban areas to the surrounding environment, animals, and plants. To evaluate the ecological risk of PAHs in PM in cities in China, we adopted a widely used ecological risk assessment method based on sediment quality guidelines (SQGs; Hui et al., 2022; Liu et al., 2020). The effect range medium (ERM) and effect range low (ERL) were compared to evaluate the ecological risks of PAHs. The concentrations of PAHs and the values of ERM and ERL are listed in Table 1. For quality evaluation, the effect range median quotient (ERM‐Q) method was used to determine the combined ecological effects of PAHs on surrounding organisms. ERM‐Q values can be divided into three classes: high risk (>1.5), medium risk (0.5–1.5), and low risk (<0.5) (Hui et al., 2022; Long et al., 1995; Wang et al., 2020). There is no minimum safety value for the four individual PAHs benzo(b)fluoranthene (BbF), benzo(k)fluoranthene (BkF), indeno(1,2,3‐cd)pyrene (IP), and benzo(g,h,i)perylene (BP). Therefore, these compounds can harm organisms and people. In addition to these four individual compounds, the contents of the other 12 individual PAHs exceeded the ERL in some urban PM samples.

**Table 1:** Number (Percent, %) of Cities Exposed to Various Ecological Risk Levels Due To Polycyclic Aromatic Hydrocarbons (PAHs) in Particulate Matter (PM)

PAH	Standards (μg/g)		Number (percent, %) of cities with the indicated ecological risks								
			NC			CC			SC		
	ERL	ERM	<ERL (%)	ERL–ERM (%)	>ERM (%)	<ERL (%)	ERL–ERM (%)	>ERM (%)	<ERL (%)	ERL–ERM (%)	>ERM (%)
Acy	1.6E−2	5.0E−1	21 (51.2%)	7 (17.1%)	13 (31.7%)	8 (26.7%)	10 (33.3%)	12 (40.0%)	4 (22.2%)	8 (44.4%)	6 (33.3%)
Fla	6.0E−1	5.1E+0	6 (14.6%)	22 (53.7%)	13 (31.7%)	25 (83.3%)	5 (16.7%)	0	13 (72.2%)	4 (22.2%)	1 (5.6%)
Nap	1.6E−1	2.1E+0	16 (39.0%)	20 (48.8%)	5 (12.2%)	18 (60.0%)	10 (33.3%)	2 (6.7%)	10 (55.6%)	7 (38.9%)	1 (5.6%)
Chr	3.8E−1	2.8E+0	3 (7.3%)	20 (48.8%)	18 (43.9%)	6 (20.0%)	23 (76.7%)	1 (3.3%)	9 (50.0%)	6 (33.3%)	3 (16.7%)
Pyr	6.7E−1	2.6E+0	2 (4.9%)	14 (34.1%)	25 (61.0%)	20 (66.7%)	10 (33.3%)	0	9 (50.0%)	4 (22.2%)	5 (27.8%)
Ace	4.4E−2	6.4E−1	11 (26.8%)	9 (22.0%)	21 (51.2%)	9 (30.0%)	12 (40.0%)	9 (30.0%)	9 (50.0%)	4 (22.2%)	5 (27.8%)
Ant	8.5E−2	1.1E+0	4 (9.8%)	11 (26.8%)	26 (63.4%)	5 (16.7%)	20 (66.7%)	5 (16.7%)	9 (50.0%)	5 (27.8%)	4 (22.2%)
Phe	2.4E−1	1.5E+0	3 (7.3%)	8 (19.5%)	30 (73.2%)	1 (3.3%)	21 (70.0%)	8 (26.7%)	10 (55.6%)	2 (11.1%)	6 (33.3%)
BaP	4.3E−1	1.6E+0	2 (4.9%)	6 (14.6%)	33 (80.5%)	2 (6.7%)	22 (73.3%)	6 (20.0%)	9 (50.0%)	4 (22.2%)	5 (27.8%)
BaA	2.6E−1	1.6E+0	1 (2.4%)	7 (17.1%)	33 (80.5%)	0	22 (73.3%)	8 (26.7%)	6 (33.3%)	9 (50.0%)	3 (16.7%)
Flu	1.9E−2	5.4E−1	12 (29.3%)	6 (14.6%)	23 (56.1%)	2 (6.7%)	12 (40.0%)	16 (53.3%)	3 (16.7%)	8 (44.4%)	7 (38.9%)
DA	6.3E−2	2.6E−1	2 (4.9%)	0	39 (95.1%)	2 (6.7%)	0	28 (93.3%)	3 (16.7%)	5 (27.8%)	10 (55.5%)
∑PAHs	4.0E+0	4.5E+1	0	10 (24.4%)	31 (75.6%)	0	27 (90.0%)	3 (10.0%)	5 (27.8%)	6 (33.3%)	7 (38.9%)

*Note.* ERL: effect range low; ERM: effect range medium (Long et al., 1995).

The ERM‐Q method was used to determine the effect of each individual PAH on the environment. The calculation formula is as follows:

(1) $$\text{ERM}-Q=\left[\sum \left(C_{i}/{\text{ERM}}_{i}\right)\right]/n$$

where *C*_*i*_ is the concentration of each individual PAH, ERM_*i*_ is the target ERM of the individual PAH, and *n* is the number of PAHs. When 0.1 ≤ ERM‐Q < 0.5, the ecological risk is low. When 0.5 < ERM‐Q ≤ 1.5, the ecological risk is considered moderate. When ERM‐Q > 1.5, the ecological risk is high.

#### 2.3.2 Benzo(a)pyrene Equivalents (BaPeq)

The TEQ is the total equivalent toxic concentration of a PAH in PM. TEQ was calculated using Equation 2 (Gao et al., 2018):

(2) $$\text{TEQ}=\Sigma \;\left(C_{i}\times {\text{TEF}}_{i}\right)$$

where *C*_*i*_ is the concentration of the individual PAH (Table S1 in Supporting Information S1), and the toxic equivalency factors of the 16 PAHs were derived from the literature (Table S2 in Supporting Information S1; Nisbet & LaGoy, 1992; Wang et al., 2019).

#### 2.3.3 Noncancer Risk

The noncarcinogenic risk resulting from exposure to a chemical is based on the ratio of the inhalation PAH concentration (*IC*_*i*_) to the reference concentration for inhalation (*RfC*_*i*_). This ratio is known as the hazard index (*HI*) and was calculated using Equation 3 (US EPA, 2011):

(3) $$HI={IC}_{i}/{RfC}_{i}$$

The *EC*_*i*_ for inhalation was estimated using Equation 4:

(4) $${IC}_{i}=C_{i}\times ET\times EF\times ED\times 10^{-6}/\left(BW\;\times \;AT\right)$$

where *IC*_*i*_ is the inhalation concentration of PAH *i*, *C*_*i*_ is the content of PAH *i* in PM, *ET* is the exposure time [inhalation time (min/d)/(60 min × 24 hr), h/d], *EF* is the exposure frequency (365, *d*/*a*), ED is the average lifetime exposure duration [6 years for children, 12 years for pupils, 18 years for teenagers, and (average lifespan—18) a for adults], *BW* is body weight, and *AT* is the exposure time for each age group (a) (Table S4 in Supporting Information S1; Duan, 2016; US EPA, 2010; Zhao & Duan, 2014). Due to significant differences in the health risk parameters (average lifespan, inhalation volume, inhalation time, body weight, etc.) of people among different regions of China, we divided people into four age groups. Detailed information on each parameter used in the different formulas is given in Table S4 in Supporting Information S1 for four age groups [children (<6), pupils (6–12), teenagers (12–18), and adults (>18)]. Additionally, a receptor may be exposed to a specific family of chemicals associated with noncarcinogenic effects. For the noncancer risk, an *HI* value > 10 indicates extremely high noncarcinogenic effects. An *HI* value > 1 the existence of non carcinogenic effects. An *HI* value < 1 indicates that adverse health impacts are unlikely (Leung et al., 2008; US EPA, 2011; Wu et al., 2020, 2024).

#### 2.3.4 Cancer Risk

The cancer risk of the urban population was calculated using the lifetime incremental cancer risk (ILCR) model. ILCR_*i*_ can be calculated as follows:

(5) $${\text{ILCR}}_{i}={\text{CSF}}_{i}\times \frac{\text{TEQ}\times IR\times EF\times ED}{BW\times AT}\times 10^{-6}$$

where CSF_*i*_ is the cancer slope factor, 3.14 (mg/kg/d)^−1^; TEQ is the total BaP equivalent concentration (*BaP*_eq_, ng/m^3^); *IR* is the inhalation rate [inhalation volume (L/min) × inhalation time (min/d)]; *EF* is the exposure frequency (365, *d*/*a*); *ED* is the exposure duration [children (6), pupils (12) and teenagers (18), adults (average lifespan), a]; *BW* is the human body weight (kg); and *AT* is the average exposure time (a). The values of these parameters are displayed in Tables S2–S4 in Supporting Information S1 (Duan, 2016; US EPA, 2010; Wu et al., 2020; Zhao & Duan, 2014).

Regarding cancer risk via inhalation, the World Health Organization (WHO, 2000) suggests a risk of 8.7 × 10^−5^ (ng/m^3^)^−1^ for PAH exposure over a lifetime of 70 years. Assuming that one unit of BaP corresponds to exposure to an average concentration of 1.0 ng/m^3^, the concentrations corresponding to a risk of 10^−6^, 10^−5^, and 10^−4^ are 0.012, 0.12, and 1.2 ng/m^3^, respectively (WHO, 2000). The ILCR is the probability of an individual developing cancer from lifetime exposure. ILCR values of 1.0E−6, 5.0E−5, and 1.0E−4 indicate low, medium and high potential cancer risks, respectively. These values are based on the assumption that one additional case in a population of 1.0 × 10^6^, 5.0 × 10^5^, or 1.0 × 10^4^ represents an acceptable, moderate, or unacceptable risk, respectively (Callén et al., 2014; Wittawat et al., 2022).

## 3 Results and Discussion

### 3.1 PAHs in PM in China

The spatial distribution of PAHs in PM in China was plotted using ArcGIS 10.2 (ESRI Co., United States of America). Figure 1a shows that high PAH concentrations occurred mainly in NC (e.g., Jilin, Fushun, Jinzhong, Tianjin, Urumqi, and Shenyang). However, low PAH concentrations were mainly distributed in CC and SC, in Hsinchu, Taichung, Hong Kong, Taipei, Macau, and Xiamen. Overall, the PAH content varied widely among the different cities. Therefore, according to the geographical latitude of NC, CC and SC, as well as the significant differences in PAHs in urban PM within and between regions, we divided the concentrations of PAHs in urban PM into three specific ranges—< 50, 50–500, and >500 ng/m^3^—for discussion and analysis. Figure 1a shows that the proportions of PAHs with a content <50.0 ng/m^3^ were 51.2%, 86.7%, and 66.7% in NC, CC, and SC, respectively. The proportions of PAHs with a content between 50.0 and 500.0 ng/m^3^ were 29.3%, 13.3%, and 33.3% in NC, CC, and SC, respectively. The proportions of PAHs with a content >500.0 ng/m^3^ were 19.5%, 0.0%, and 0.0% in NC, CC, and SC, respectively.

The highest and lowest PAH average concentrations were 1,201.4 ng/m^3^ in Jilin and 1.6 ng/m^3^ in Hsinchu, respectively (Table S1 in Supporting Information S1; Chen et al., 2014; Zhang, 2012). PAH pollution in PM also occurred in other cities to a certain degree (Figure 1b). High mean PAH values (ng/m^3^) occurred in the cities of Fushun (1,081.1), Jinzhong (910.5), and Tianjin (865.8). Low mean PAH values (ng/m^3^) occurred in Taichung (2.1), Hong Kong (2.5), and Taipei (2.5). Overall, the mean PAH concentrations were 241.3, 33.0, and 54.0 ng/m^3^ in NC, CC, and SC, respectively. The PAH content decreased in the order of NC > SC > CC. In NC, industrial and residential heating is necessary due to the long winter (Li et al., 2005). Coal‐fired heating in winter is the main source of PAHs in northern cities. For example, 37.5% of PAHs in Beijing's Changping urban area come from coal sources, and the concentration in winter is significantly higher than that in other seasons (Zhang, 2019). Industrial activities (such as steel and coking) contribute significantly to emissions; for example, industrial emissions from coal/combustion account for 31.3% of PAH sources in Jinan city (Liu et al., 2024). PAHs are produced by the use of coal or biomass fuel in textile and paper production and other light industries and are discharged into the atmosphere in the form of PM (Xia et al., 2024). PAH emissioned from machinery manufacturing, especially in economically developed southeast coastal provinces (such as Guangdong and Jiangsu) (Li et al., 2021). The sources of PAHs in PM in Shenzhen include motor vehicle exhaust and marine and industrial emissions (Cai et al., 2020). The number of motor vehicles in southern cities such as Guangzhou was large, and the proportion of PAHs in exhaust emissions was high, especially in the central city (Feng et al., 2024). The observed trends in PAH contents were also related to the intensive oil processing, iron and steel smelting, and heating activities in NC (Zhang, 2011), while light industry (electrical appliance, electronics, and textile manufacturing), machinery manufacturing, and related industries are the primary emission sources in the warm CC and SC regions (Yan et al., 2019).

**Figure 2:**
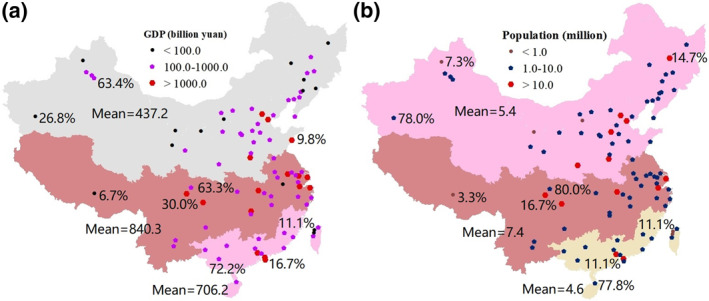
Gross domestic product (GDP) and population in North China (NC), Central China (CC), and South China (SC). (a) GDP in Chinese cities. The black circles denote a GDP of <100.0 billion yuan (observed in 26.8%, 6.7%, and 11.1% of the cities in NC, CC, and SC, respectively). The purple pentagons denote a GDP of 100.0–1,000.0 billion yuan (observed in 63.4%, 63.3%, and 72.2% of cities in NC, CC, and SC, respectively). The red hexagons denote a GDP of >1,000.0 billion yuan (observed in 9.8%, 30.0%, and 16.7% of cities in NC, CC, and SC, respectively). The gray, yellow, and red areas indicate NC, CC, and SC, respectively, which had mean GDP values of 437.2, 840.3, and 706.2 billion yuan. (b) Population in Chinese cities. The orange circles denote a population of <1.0 million people (observed in 7.3%, 3.3%, and 11.1% of cities in NC, CC, and SC, respectively). The blue pentagons denote a population of 1.0–10.0 million people (observed in 78.0%, 80.0%, and 77.8% of cities in the corresponding three regions). The red hexagons denote a population of >10.0 million people (observed in 14.7%, 16.7%, and 11.1% of cities in the corresponding three regions). The red, green, and yellow areas indicate NC, CC, and SC, respectively, which had mean populations of 5.4, 7.4, and 4.6 million people.

The relationships between the population and gross domestic product (GDP, Statistical Bulletin on National Economic and Social Development in 2019 for each city) and the PAH concentrations in PM in Chinese cities were also evaluated. As shown in Figure 2a and Table S1 in Supporting Information S1, the regions in China were ranked by GDP (10^9^ yuan) as follows: NC (437.2) < SC (706.2) < CC (840.3). The proportions of cities with a GDP of <100.0 billion yuan were 26.8%, 6.7%, and 11.1% in NC, CC, and SC, respectively. The proportions of cities with a GDP of 100–1,000 billion yuan were 63.4%, 63.3%, and 72.2% in NC, CC, and SC, respectively. The proportions of cities with a GDP of >1,000.0 billion yuan were 9.8%, 30.0%, and 16.7% in NC, CC, and SC, respectively. The regions in China were ranked by population (million people) as follows: SC (4.6) < NC (5.4) < CC (7.4) (Figure 2b; Table S1 in Supporting Information S1). The proportions of cities with a population of <1.0 million were 7.3%, 3.3%, and 11.1% in NC, CC, and SC, respectively. The proportions of cities with a population of 1.0–10.0 million people were 78.0%, 80.0%, and 77.8% in NC, CC, and SC, respectively. The proportions of cities with a population of >10.0 million were 14.7%, 16.7%, and 11.1% in NC, CC, and SC, respectively. In NC, although the per capita GDP (thousand yuan/person) was lower than those in SC and CC, the PAH concentrations (ng/m^3^) per unit GDP (10^10^ yuan) and per capita were higher than those in CC and SC. The PAH concentrations in PM across the regions of China indicated more severe PAH pollution in NC (where the predominant sources are coal and biomass) than in SC and CC, and GDP was significantly lower in NC (Li et al., 2005; Zhang, 2011). In comparison, in CC and SC, the types of industry (light industry or machinery manufacturing) may be the main factor causing the significantly higher GDPs and lower PAH pollution levels (Yan et al., 2019; Zhang et al., 2016). Overall, objective conditions [such as latitude (Pearson correlation coefficient 0.435, Table S6 in Supporting Information S1), geography, and climate] were closely related to PAH content, while GDP (−0.162) and population (−0.068) were only weakly correlated with PAH content. Therefore, further investigation of the ecological risk of PAHs was needed.

### 3.2 Ecological Risk of PAHs in PM in China

In NC, the acenaphthylene (Acy) concentration was below the ERL in 51.2% of the cities (Table 1), representing a low ecological risk and possibly originating from natural sources. Fluoranthene (Fla), naphthalene (Nap), and chrysene (Chr) concentrations were in the ERL to ERM range in 53.7%, 48.8%, and 48.8% of the cities, respectively. These three PAHs in NC represented a medium ecological risk and could come from oil combustion sources (Sobhanardakani, 2017, 2018; Sobhanardakani et al., 2018). The concentrations of the other eight PAHs were above the ERM in 51.2%–95.1% of the cities. These PAHs posed a high ecological risk and might come from coal and biomass combustion sources (Bai et al., 2023; Wu et al., 2020, 2024). For example, the main sources of PAHs in Beijing, Tianjin, Shanghai, Xi 'an and other cities include coal combustion, vehicle exhaust emissions, industrial emissions and biomass combustion, with significant variations with respect to season: the concentration of PAHs is high in the winter and nonheating periods (Shi et al., 2020). Overall, PAH concentrations in NC were closely related to biomass burning and coal burning (Zhang, 2010).

In CC, Fla, Nap, and pyrene (Pyr) concentrations were below the ERL in 83.3%, 60.0%, and 66.7%, respectively, of the cities (Table 1). These three PAHs represented a low ecological risk and might come from natural sources (Jiang et al., 2023; Li et al., 2024). Acy, fluorene (Flu), and dibenzo(a, h)anthracene (DA) concentrations were above the ERM in 40.0%, 53.3%, and 93.3% of the cities, respectively. These three PAHs represented a high ecological risk and could come from fossil fuel combustion sources (Dai et al., 2024; Gu et al., 2023). The concentrations of the other six PAHs were in the ERL to ERM range in 40.0%–76.7% of the cities. These six PAHs posed a medium level of ecological risk and might be derived from oil combustion sources (Bai et al., 2023; Wu et al., 2020). For example, in the urban area of Wuhan, vehicle exhaust emissions (34%) and natural gas combustion (25%) had high contributions (Zhou et al., 2013). The contribution of wood combustion to PAHs in CC was 46% (Zhou et al., 2013). Light industry and machinery manufacturing (such as the building materials, metal processing and other industries) might contribute to PAH emissions through localized production chains (Xia et al., 2024). In Nanjing, motor vehicle exhaust emissions were the main sources of PAH emissions, followed by natural gas combustion, high‐temperature incineration, coal burning and cooking emissions (Meng et al., 2015).

**Figure 3:**
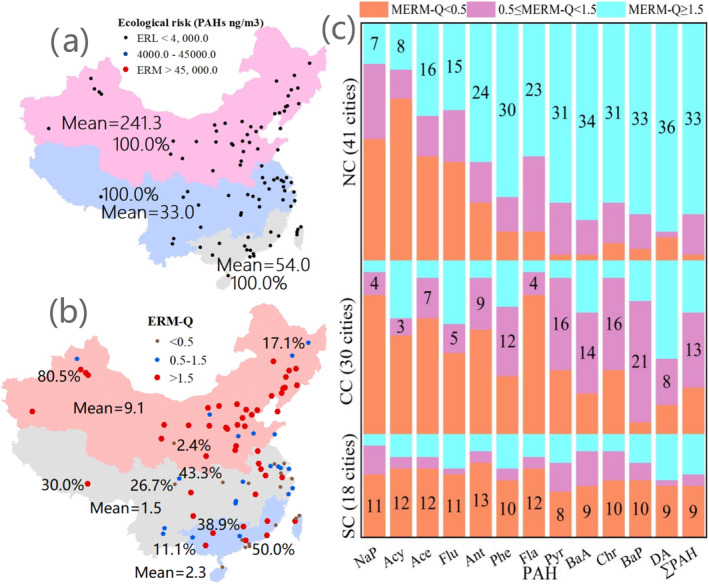
Ecological risks of polycyclic aromatic hydrocarbons (PAHs) in the three regions. (a) Ecological risks in North China (NC), Central China (CC), and South China (SC). The black circles denote an ecological risk (∑PAHs, ng/m^3^) corresponding to an effect range low (ERL) <4,000 ng/m^3^. The ecological risks in cities nationwide were lower than the ERL. (b) Proportions of city‐level effect range median quotient (ERM‐Q) values in different ranges in NC, CC, and SC. The circles denote an ERM‐Q < 0.5 (observed in 2.4%, 26.7%, and 50.0% of the cities in the above three regions). The blue pentagons denote 0.5 < ERM‐Q < 1.5 (observed in 17.1%, 43.3%, and 11.1% of the cities in the above three regions). The red hexagons denote an ERM‐Q > 1.5 (observed in 80.5%, 30.0%, and 38.9% of the cities in the above three regions). The red, gray, and blue areas indicate NC, CC, and SC, respectively, which had mean ERM‐Q values of 9.1, 1.5, and 2.3. (c) City‐level ERM‐Q values in NC, CC, and SC. The orange columns denote cities with ERM‐Q < 0.5 in the above three regions. The pink columns denote cities with 0.5 < ERM‐Q < 1.5 in the above three regions. The light blue columns denote cities with ERM‐Q > 1.5 in the above three regions.

In SC, the Acy, benzo(a)anthracene (BaA), and Flu concentrations were in the ERL to ERM range in 44.4%, 50.0%, and 44.4% of the cities, respectively (Table 1). These three PAHs presented a medium ecological risk and might come from fossil fuel combustion sources (Gu et al., 2023; Li et al., 2024). The DA concentration was above the ERM in 55.5% of the cities. This compound posed a high ecological risk and could come from fossil fuel combustion sources (Dai et al., 2024; Jiang et al., 2023). The concentrations of the other eight PAHs were lower than the ERL in 50.0%–72.2% of the cities. These eight PAHs posed a low ecological risk and might come from natural sources (Bai et al., 2023; Wu et al., 2020, 2024). The main sources of PAHs in PM in Guangzhou's atmosphere were motor vehicle exhaust and coal burning, of which motor vehicles constituted the main pollution source, accounting for 69%, followed by coal burning, accounting for 31% (Li et al., 2004). PAHs in Pearl River Delta cities are closely related to industrial zoning (Niu et al., 2024). Regarding the total amount of 12 PAHs (∑_12_PAHs), their ecological risk in all the cities was lower than the ERL (Figure 3a).

As coexisting PAHs may cause different effects than individual PAHs and because calculating the ecological risk due to simultaneous exposure to a mixture of PAHs based on the risks of the individual PAHs may overestimate or underestimate the actual risk (Bai et al., 2023; Wu et al., 2020), the ERM‐Q method was used to evaluate the combined ecological risks of the 16 priority PAHs in Chinese cities (Table 2). In NC, most individual PAHs exhibited high‐risk concentrations, with this being the case for 80.5% of the cities (high ecological risk, Figure 3b). In CC, most individual PAHs exhibited medium‐risk concentrations, which was the case for 43.3% of the cities (medium ecological risk). In SC, most individual PAHs exhibited low‐risk concentrations, which was the case for 50.0% of the cities (low ecological risk). Across China, the ecological risks of PAHs were low, moderate, and high in 18, 22, and 49 cities, respectively (Table 2 and Table S7 in Supporting Information S1, Figure 3c). A total of 97.6%, 73.3%, and 50.0% of the cities in NC, CC, and SC, respectively, had an ERM‐Q > 0.5. These results are very similar to those obtained for India in South Asia (Arushi et al., 2023; Kunal et al., 2021; Wu et al., 2020, 2024).

**Table 2:** Number (Percent, %) of Cities With the Indicated Effect Range Median Quotient (ERM‐Q) Values

PAH	ERM	Number of cities with the indicated ERM‐Q (percent, %)								
		NC			CC			SC		
		<0.5 (%)	0.5–1.5 (%)	≥1.5 (%)	<0.5 (%)	0.5–1.5 (%)	≥1.5 (%)	<0.5 (%)	0.5–1.5 (%)	≥1.5 (%)
Nap	2.1E−6	21 (51.2%)	13 (31.7%)	7 (17.1%)	24 (80.0%)	4 (13.3%)	2 (6.7%)	11 (61.2%)	5 (27.7%)	2 (11.1%)
Acy	5.0E−7	28 (68.3%)	5 (12.2%)	8 (19.5%)	17 (56.7%)	3 (10.0%)	10 (33.3%)	12 (66.7%)	2 (11.1%)	4 (22.2%)
Ace	6.4E−7	18 (43.9%)	7 (17.1%)	16 (39.0%)	20 (66.7%)	7 (23.3%)	3 (10.0%)	12 (66.7%)	2 (11.1%)	4 (22.2%)
Flu	5.4E−7	17 (41.5%)	9 (22.0%)	15 (36.6%)	14 (46.7%)	5 (16.7%)	11 (36.6%)	11 (61.2%)	1 (5.6%)	6 (33.3%)
Ant	1.1E−6	10 (24.4%)	7 (17.1%)	24 (58.5%)	18 (60.0%)	9 (30.0%)	3 (10.0%)	13 (72.2%)	2 (11.1%)	3 (16.7%)
Phe	1.5E−6	5 (12.2%)	6 (14.6%)	30 (73.2%)	10 (33.3%)	12 (40.0%)	8 (26.7%)	10 (55.5%)	2 (11.1%)	6 (33.3%)
Fla	5.1E−6	5 (12.2%)	13 (31.7%)	23 (56.1%)	24 (80.0%)	4 (13.3%)	2 (6.7%)	12 (66.7%)	2 (11.1%)	4 (22.2%)
Pyr	2.6E−6	1 (2.4%)	9 (22.0%)	31 (75.6%)	11 (36.7%)	16 (53.3%)	3 (10.0%)	8 (44.4%)	5 (27.8%)	5 (27.8%)
BaA	1.6E−6	1 (2.4%)	6 (14.6%)	34 (82.9%)	7 (23.3%)	14 (46.7%)	9 (30.0%)	9 (50.0%)	6 (33.3%)	3 (16.7%)
Chr	2.8E−6	3 (7.3%)	7 (17.1%)	31 (75.6%)	11 (36.6%)	16 (53.4%)	3 (10.0%)	10 (55.5%)	5 (27.8%)	3 (16.7%)
BaP	1.6E−6	2 (4.9%)	6 (14.6%)	33 (80.5%)	2 (6.7%)	21 (70.0%)	7 (23.3%)	1 (5.6%)	3 (16.7%)	5 (27.8%)
DA	2.6E−7	4 (9.8%)	1 (2.4%)	36 (87.8%)	5 (16.7%)	8 (26.7%)	17 (56.7%)	9 (50.0%)	1 (5.6%)	8 (44.4%)
∑PAHs	1.7E−6	1 (2.4%)	7 (17.1%)	33 (80.5%)	8 (26.7%)	13 (43.3%)	9 (30.0%)	9 (50.0%)	2 (11.1%)	7 (38.9%)

### 3.3 Composition and Toxicity Equivalent Concentrations of PAHs in PM

**Figure 4:**
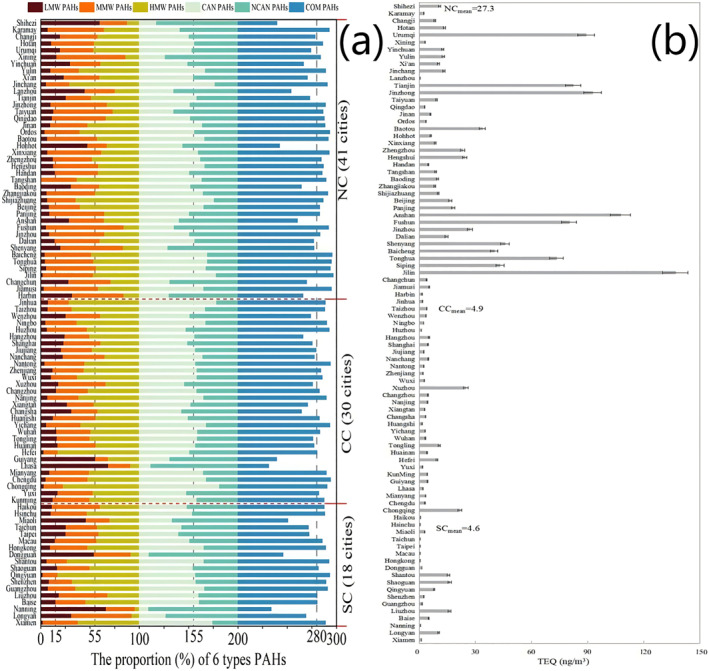
Composition of polycyclic aromatic hydrocarbon (PAH) and TEQ. (a) Proportions (%) of six types of PAHs. The brown columns indicate the proportion of LMW PAHs (∼15%). The orange columns denote the proportion of MMW PAHs (∼40%). The yellow columns denote the proportion of HMW PAHs (∼45%). The light green columns denote the proportion of CAN‐PAHs (∼55%). The dark green columns denote the proportion of NCAN‐PAHs (∼45%). The blue columns denote the proportion of COM‐PAHs (∼80%). (b) TEQs of PAHs in particulate matter in 89 cities. The TEQs in most cities are greater than 1.0. The mean TEQs are 27.3, 4.9, and 4.6 in North China, Central China, and South China, respectively.

The contributions of congeners of low‐molecular‐weight PAHs (LMW‐PAHs, including Ace, Acy, Ant, Flu, Nap, and Phe), medium‐molecular‐weight PAHs (MMW‐PAHs, including BaA, Chr, Fla, and Pyr) and high‐molecular‐weight PAHs (HMW‐PAHs, including BaP, BbF, BkF, BP, DA, and IP) described by Li et al. (2016) and Wang et al. (2019) were similar. As shown in Figure 4a, LMW‐, MMW‐ and HMW‐PAHs accounted for 15%, 40%, and 45%, respectively, of Σ_16_PAHs (Table S8 in Supporting Information S1). Carcinogenic PAHs (CAN‐PAHs, including BaA, BbF, BkF, BaP, Chr, DA, and IP) accounted for ∼55% of Σ_16_PAHs. Noncarcinogenic PAHs (NCAN‐PAHs, Ace, Acy, Ant, BP, Fla, Flu, Nap, Phe, and Pyr) accounted for ∼45% of Σ_16_PAHs. Combustion‐derived PAHs (COM‐PAHs; BaA, BaP, BbF, BkF, BP, Chr, Fla, IP, Pyr) accounted for ∼80.0% of Σ_16_PAHs (Li et al., 2016; Wang et al., 2019). Although the urban PAH composition might differ depending on location, this might be partly due to differences in pollution sources, emissions and characteristics (Arushi et al., 2023; Kunal et al., 2021). NC was dominated by coal, fuel oil, biomass, and natural gas sources. CC was dominated by biomass and fuel oil sources. SC was dominated by electric energy and new energy sources (Fu et al., 2009; Xiao et al., 2014; Yu et al., 2014; Zhang et al., 2013).

According to WHO standards (2000), in NC, no city exhibited a low risk (TEQ <0.012 ng/m^3^), only Lanzhou city (2.4%) had a medium risk (0.012 < TEQ < 1.2 ng/m^3^), and 40 cities (97.6%) demonstrated a high risk (TEQ > 1.2 ng/m^3^) (Figure 4b). In CC, all the cities had a high risk. In SC, seven cities (38.9%) belonged to the medium‐risk category, and 11 cities (61.1%) belonged to the high‐risk category. Among the 89 cities, 8 cities (9.0%) were classified as exhibiting a medium risk, and 81 (91.0%) were classified as exhibiting a high risk. The mean TEQ in NC (27.3 ng/m^3^) was significantly greater than those in CC (4.9 ng/m^3^) and SC (4.6 ng/m^3^). Therefore, it is necessary to analyze the health risk of PAHs in PM to the populations in these cities.

### 3.4 Noncancer Risk of PAHs in PM in China

**Figure 5:**
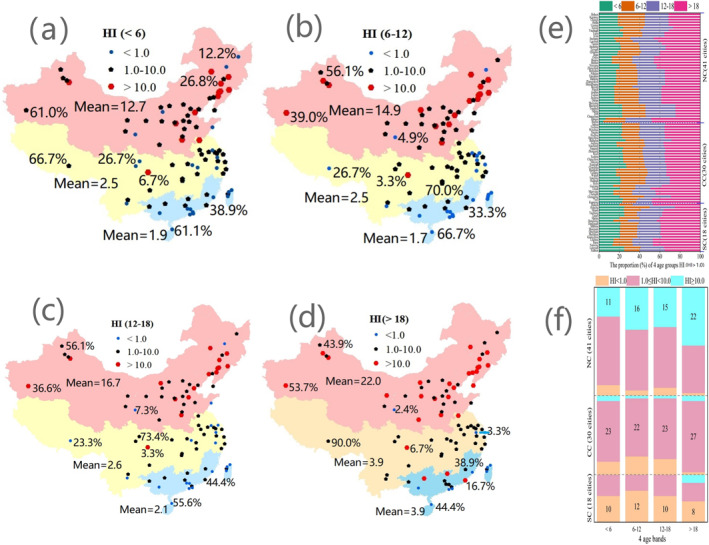
HIs for Chinese urban residents. (a) hazard index (HI) for children (<6). The blue circles denote HI < 1.0 (observed in 12.2%, 26.7%, and 61.1% of the cities in North China (NC), Central China (CC), and South China (SC), respectively). The black pentagons denote 1.0 < HI < 10.0 (observed in 61.0%, 66.7%, and 38.9% of the cities in the above three regions). The red hexagons denote HI > 10.0 (observed in 26.8%, 6.7%, and 0.0% of the cities in the above three regions). The red, yellow, and blue areas indicate NC, CC, and SC, respectively, which had mean HIs of 12.7, 2.5, and 1.9. (b) HI for pupils (aged 6–12). The blue circles denote HI < 1.0 (observed in 4.9%, 26.7%, and 66.7% of the cities in NC, CC, and SC, respectively). The black pentagons denote 1.0 < HI < 10.0 (observed in 56.1%, 70.0%, and 33.3% of the cities in the above three regions). The red hexagons denote HI > 10.0 (observed in 39.0%, 3.3%, and 0.0% of the cities in the above three regions). The red, yellow, and blue areas indicate NC, CC, and SC, respectively, which had mean HI values of 14.9, 2.5, and 1.7. (c) HI for teenagers (aged 12∼18). The blue circles denote HI < 1.0 (observed in 7.3%, 23.3%, and 55.6% of the cities in NC, CC, and SC, respectively). The black pentagons denote 1.0 < HI < 10.0 (observed in 56.1%, 73.4% and 44.4% of the cities in the above three regions). The red hexagons denote HI > 10.0 (observed in 36.6%, 3.3%, and 0.0% of the cities in the above three regions). The red, yellow, and blue areas indicate NC, CC, and SC, respectively, which had mean HI values of 16.7, 2.6, and 2.1. (d) HI for adults (>18). The blue circles denote HI < 1.0 (observed in 2.4%, 3.3%, and 44.4% of the cities in NC, CC, and SC, respectively). The black pentagons denote 1.0 < HI < 10.0 (observed in 43.9%, 90.0%, and 38.9% of the cities in the above three regions). The red hexagons denote HI > 10.0 (observed in 53.7%, 6.7%, and 16.7% of the cities in the above three regions). The red, yellow, and blue areas indicate NC, CC, and SC, respectively, which had mean HI values of 22.0, 3.9, and 3.9. (e) Proportions (%) of the four age groups with an HI > 1.0 in the three regions. The proportions (%) of the age groups of <6, 6∼12, 12∼18, and >18 years with an HI > 1.0 were approximately 20%, 20%, 20%, and 40%, respectively. (f) Number of cities in the three regions with HI values in the indicated intervals for the four age groups. Most cities in SC, CC, and NC presented values of <1.0, 1.0 < HI < 10.0, and HI > 10.0, respectively.

Here, we evaluated the noncarcinogenic risks of PAHs in PM via inhalation (Table S9 in Supporting Information S1). For children (<6, Figure 5a), the lowest and highest HIs for PAHs were observed in NC, with values of 0.1 (Harbin and Lanzhou) and 46.1 (Jilin), respectively, and a mean value of 12.7 (>10.0, extremely high chronic risk level; Leung et al., 2008). The HIs for PAHs in 36 cities were above 1.0. Among them were 25 cities with HIs of 1.0–10.0 and 11 cities with HIs over 10.0, accounting for 61.0% and 26.8%, respectively, of the 41 cities in NC (Table 3). In CC, the lowest and highest risks were 0 (Lhasa) and 10.3 (Chongqing), respectively, with a mean value of 2.5. The HIs for PAHs in 22 cities were greater than 1.0. Among them were 20 cities with HIs of 1.0–10.0 and two cities with HIs over 10.0, accounting for 66.7% and 6.7%, respectively, of the 30 cities in CC. In SC, the lowest and highest risks were 0 (Hsinchu) and 8.0 (Liuzhou), respectively, with a mean value of 1.9. The HI values for PAHs in seven cities in SC ranged from 1.0 to 10.0, representing 38.9% of the 18 cities in SC. No cities had an HI > 10.0. Overall, the HI values of 13 cities (Jilin, Siping, Tonghua, Baicheng, Fushun, Anshan, Hengshui, Zhengzhou, Jinzhong, Tianjin, and Urumqi in NC; Chongqing and Xuzhou in CC) exceeded 10.0. Therefore, the HI was significantly high for children in cities in China. The increasing order of HI in the three regions was SC < CC < NC.

**Table 3:** Number (Percent, %) of Cities in the Three Regions With the Indicated Hazard Index (HI) and Lifetime Incremental Cancer Risk (ILCR) Values for the Four Age Groups

Age groups	HI	Number (%) of cities in NC	Number (%) of cities in CC	Number (%) of cities in SC	ILCR	Number (%) of cities in NC	Number (%) of cities in CC	Number (%) of cities in SC
<6	<1.0	5 (12.2%)	8 (26.7%)	11 (61.1%)	<1.0E−6	3 (7.3%)	41 (100%)	41 (100%)
	1.0–10.0	25 (61.0%)	20 (66.7%)	7 (38.9%)	1.0E−6‐5.0E−5	38 (92.7%)	0	0
	>10.0	11 (26.8%)	2 (6.7%)	0	>5.0E−5	0	0	0
6–12	<1.0	2 (4.9%)	8 (26.7%)	12 (66.7%)	<1.0E−6	6 (14.6%)	41 (100%)	41 (100%)
	1.0–10.0	23 (56.1%)	21 (70.0%)	6 (33.3%)	1.0E−6‐5.0E−5	35 (85.4%)	0	0
	>10.0	16 (39.0%)	1 (3.3%)	0	>5.0E−5	0	0	0
12–18	<1.0	3 (7.3%)	7 (23.3%)	10 (55.6%)	<1.0E−6	9 (22.0%)	41 (100%)	41 (100%)
	1.0–10.0	23 (56.1%)	22 (73.4%)	8 (44.4%)	1.0E−6–5.0E−5	33 (78.0%)	0	0
	>10.0	15 (36.6%)	1 (3.3%)	0	>5.0E−5	0	0	0
>18	<1.0	1 (2.4%)	1 (3.3%)	8 (44.4%)	<1.0E−6	16 (39.0%)	26 (86.7%)	15 (83.3%)
	1.0–10.0	18 (43.9%)	27 (90.0%)	7 (38.9%)	1.0E−6–5.0E−5	25 (61.0%)	4 (13.3%)	3 (16.7%)
	>10.0	22 (53.7%)	2 (6.7%)	3 (16.7%)	>5.0E−5	0	0	0

For pupils (aged 6–12), the HIs for PAHs in 39 cities in NC were greater than 1.0 (Figure 5b). Among them, there were 23 cities with HIs of 1.0–10.0 and 16 cities with HIs over 10.0, representing 56.1% and 39.0%, respectively, of the 41 cities in NC (Table 3). The HIs for PAHs in 22 cities in CC were also over 1.0. Among them, 21 cities had HI values of 1.0–10.0, and only Chongqing city had an HI greater than 10.0, accounting for 70.0% and 3.3%, respectively, of the 30 cities in NC. The HI values for PAHs in six cities in SC ranged from 1.0 to 10.0, representing 33.3% of the 18 cities in SC. No city had an HI exceeding 10.0.

For teenagers (12–18), the HI values for PAHs in 38 cities in NC were greater than 1.0 (Figure 5c). Among them, there were 23 cities with HIs of 1.0–10.0 and 15 cities with HIs over 10.0, representing 56.1% and 36.6%, respectively, of the 41 cities in NC (Table 3). The HI values for PAHs in 23 cities in CC were also over 1.0. Among them, 22 cities had HIs of 1.0–10.0, and only Chongqing city had an HI greater than 10.0, representing 73.4% and 3.3%, respectively, of the 30 cities in CC. The HI values for PAHs in eight cities in CC ranged from 1.0 to 10.0, representing 44.4% of the 18 cities in SC. No city had an HI exceeding 10.0.

For adults (>18), the HI values for PAHs in 40 cities in NC were greater than 1.0 (Figure 5d). Among them, 18 cities had HIs of 1.0–10.0, and 22 cities had HIs over 10.0, representing 43.9% and 53.7%, respectively, of the 41 cities in NC (Table 3). The HI values for PAHs in 29 cities in CC were also over 1.0. Among them were 27 cities with HIs of 1.0–10.0 and two cities with HIs over 10.0, representing 90.0% and 6.7%, respectively, of the 30 cities in CC. The HI values for PAHs in 10 cities in SC were also over 1.0. Among them, seven cities had HIs of 1.0–10.0, and three cities had HIs over 10.0, representing 38.9% and 16.7%, respectively, of the 18 cities in SC. In NC, the mean HIs were 12.7, 14.9, 16.7, and 22.0 for children, pupils, teenagers, and adults, respectively (Table S9 in Supporting Information S1). In CC, the mean HIs were 2.5, 2.5, 2.6, and 3.9 for the above four age groups, respectively. In SC, the mean HIs were 1.9, 1.7, 2.1, and 3.9 for the above four age groups, respectively. Overall, the lowest and highest HIs were 0 and 76.3, 0 and 88.2, 0 and 99.0, and 0.1 and 103.5, with mean values of 7.2, 8.3, 9.2, and 12.5 for children (<6), pupils (6–12), teenagers (12–18), and adults (>18), respectively. The HI values indicate possible adverse health impacts for the above four age groups in 65, 67, 69, and 79 cities, respectively (US EPA, 1993). HIs >1.0 occurred in 73.0%, 75.3%, 78.7%, and 88.8% of the cities for the above four age groups, respectively. The proportions of HIs with a value >1.0 were ∼20.0%, ∼20.0%, ∼20.0%, and ∼40.0% for the above four age groups, respectively (Figure 5e). In NC, CC, and SC, most cities had HI values in the range of HI > 10.0, 1.0 < HI < 10.0, and HI < 1.0, respectively (Figure 5f). Therefore, the HIs of PAHs can be considered high in Chinese cities. Further analysis of the cancer risk posed by PAHs in PM is needed.

### 3.5 Cancer Risk of PAHs in PM in China

**Figure 6:**
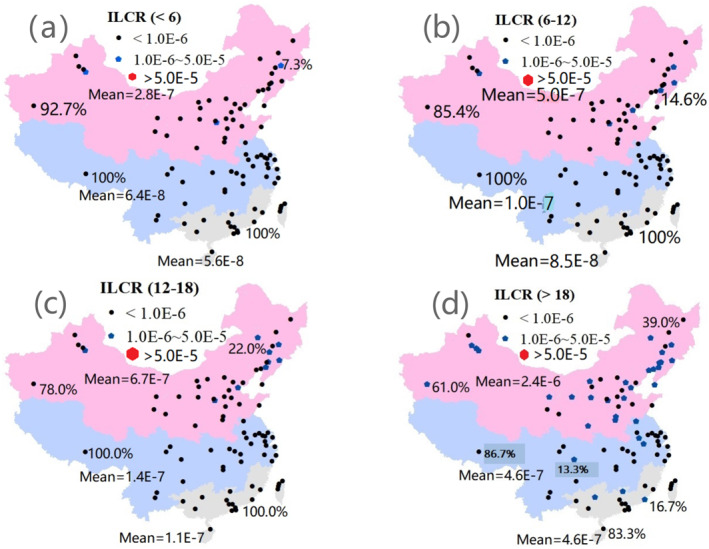
Lifetime incremental cancer risk (ILCR) for Chinese urban residents. (a) ILCR for children (<6). The black circles denote children with an ILCR < 1.0E−6 (observed in 92.7%, 100.0%, and 100.0% of cities in North China (NC), Central China (CC), and South China (SC), respectively). The blue pentagons denote 1.0E−6 < ILCR < 5.0E−5 (observed in 7.3%, 0.0%, and 0.0% of the cities in the above three regions). The red hexagons denote ILCR > 5.0E−5; there was no city with this value in the above three regions. The red, blue, and gray areas indicate NC, CC, and SC, respectively, which had mean ILCR values of 2.8E−7, 6.4E−8, and 5.6E−8. (b) ILCR for pupils (aged 6–12). The black circles denote pupils with ILCR < 1.0E−6 (observed in 85.4%, 100.0%, and 100.0% of cities in the NC, CC, and SC, respectively). The blue pentagons denote 1.0E−6 < ILCR < 5.0E−5 (observed in 14.6%, 0.0%, and 0.0% of the cities in the above three regions). The red hexagons denote ILCR > 5.0E−5; there was no city with this value in any of the three regions. The red, blue, and gray areas indicate NC, CC, and SC, respectively, which had mean ILCR values of 5.0E−7, 1.0E−7, and 8.5E−8. (c) ILCR for teenagers (aged 12–18). The black circles denote teenagers with ILCR < 1.0E−6 (observed in 78.0%, 100.0%, and 100.0% of cities in NC, CC, and SC, respectively). The blue pentagons denote 1.0E−6 < ILCR < 5.0E−5 (observed in 22.0%, 0.0%, and 0.0% of the cities in the above three regions). The red hexagons denote ILCR > 5.0E−5; there was no city with this value in any of the three regions. The red, blue, and gray areas indicate NC, CC, and SC, respectively, which had mean ILCR values of 6.7E−7, 1.4E−7, and 1.1E−7. (d) ILCR for adults (>18). The black circles represent adults with ILCR < 1.0E−6 (observed in 39.0%, 86.7%, and 83.3% of cities in NC, CC, and SC, respectively). The blue pentagons denote 1.0E−6 < ILCR < 5.0E−5 (observed in 61.0%, 13.3%, and 16.7% of the cities in the above three regions). The red hexagons denote ILCR > 5.0E−5; there was no city with this value in any of the three regions. The red, blue, and gray areas indicate NC, CC, and SC, respectively, which had mean ILCR values of 2.4E−6, 4.6E−7, and 4.6E−7.

We also evaluated the cancer risks of PAHs in PM via inhalation (Table S10 in Supporting Information S1). Figure 6 shows the ILCRs for PAHs in the four considered age groups. For children (<6, Figure 6a), the lowest and highest ILCRs of PAHs in NC were 3.0E−9 (Harbin) and 1.6E−6 (Jilin), respectively, with a mean value of 2.8E−7 (<1.0E−6, low cancer risk; Leung et al., 2008). The ILCR values of PAHs in three cities (Jilin, Jinzhong, and Urumqi) were above 1.0E−6, representing 7.3% of the 41 cities in NC (Table 3). In CC, the lowest and highest risk factors were 2.9E−8 (Lhasa) and 3.5E−7 (Chongqing), respectively, with a mean value of 6.4E−8. No cities had an ILCR >1.0E−6. In SC, the lowest and highest risks were 1.7E−9 (Hsinchu) and 2.2E−7 (Liuzhou), respectively, with a mean value of 5.6E−8. No cities had an ILCR >1.0E−6. Therefore, the ranking of ILCRs for the three regions was SC < CC < NC.

For pupils (6–12), the ILCRs of PAHs in six cities in NC were greater than 1.0E−6, accounting for 14.6% of the 41 cities in NC (Table 3). No cities in CC or SC exhibited an ILCR >1.0E−6 (Figure 6b). In contrast to the results for pupils, for teenagers (12–18), the ILCRs of PAHs in nine cities in NC exceeded 1.0E−6 (Figure 6c; Table S10 in Supporting Information S1). No cities in CC or SC had an ILCR >1.0E−6. For adults (>18), the ILCRs of PAHs in 25 cities in NC were greater than 1.0E−6, representing 61.0% of the 41 cities (Figure 6d; Table 3). The ILCRs of PAHs in four and three cities in CC and SC were greater than 1.0E−6, representing 13.3% and 16.7% of the cities in these two regions, respectively.

In NC, the mean ILCRs were 2.8E−7, 5.0E−7, 6.7E−7, and 2.4E−6 for children (<6), pupils (6–12), teenagers (12–18), and adults (>18), respectively. In CC, the mean ILCRs were 6.4E−8, 1.0E−7, 1.4E−7, and 4.6E−7 for the above four age groups, respectively. In SC, the mean ILCRs were 5.6E−8, 8.5E−8, 1.1E−7 and 4.6E−7 for the above four age groups, respectively (Figures 6a–6d). Overall, the lowest and highest ILCRs were 1.7E−9 and 1.6E−6, 2.6E−9 and 2.9E−6, 3.5E−9 and 3.9E−6, and 1.5E−8 and 1.2E−5, with mean values of 1.6E−7, 2.8E−7, 3.8E−7, and 1.4E−6 for the above four age groups, respectively (Table S10 in Supporting Information S1). According to the ILCR results, 3, 6, 9, and 49 cities are characterized by adverse health impacts for the above four age groups, respectively (US EPA, 1993). Among 89 cities across the country, the proportions of cities with ILCR >1.0E−6 in the four age groups were 3.4% (<6), 6.7% (6–12), 10.1% (12–18), and 36.0% (>18). Therefore, the ILCR of PAHs can also be considered higher in cities in China than in other cities worldwide.

Overall, the HI and ILCR values for the four age groups in ascending order were as follows: children (<6) < pupils (6–12) < teenagers (12–18) < adults (>18) (Table S10 in Supporting Information S1). Therefore, the health risks (ILCR and HI) of PAHs for adults were greater than those for juveniles. These results imply that urban residents in China, especially adults, are subject to health risks (ILCR and HI) due to PAHs in PM.

### 3.6 Discussion, Strengths and Limitations of This Study

In this study, the health risk resulting from PAHs in PM was high for urban residents. Our results are exactly the opposite of those of other studies (Arushi et al., 2023; Bai et al., 2023; Kunal et al., 2021; Sun et al., 2021; Yan et al., 2019). In those studies, children or teenagers exhibited greater health risks resulting from exposure to PAHs in PM than did adults (Sobhanardakani, 2018; Sobhanardakani et al., 2018). Our findings could be attributed to the longer exposure time and higher respiratory rate of adults resulting in a higher cumulative health risk than those of children or teenagers. Moreover, the available research mostly shows that PAHs in PM pose a high health risk for urban residents (Bai et al., 2023; Kunal et al., 2021; Sun et al., 2021).

Therefore, Chinese cities should vigorously implement PAH reduction strategies. Potential strategies could include the following: (a) Optimize the energy structure by promoting clean energy, such as natural gas, solar energy, wind energy, and geothermal energy; improving energy utilization efficiency; and promoting the adoption of energy‐saving windows, wall insulation materials, etc. Strengthen pollution source control by strengthening supervision of industrial enterprises that emit PAHs, such as coal‐fired power plants, aluminum smelters, and coking plants; strengthening supervision of residential combustion of garbage and straw; and strictly prohibiting open‐air burning. Control traffic pollution sources by promoting the use of new energy vehicles; encouraging residents to purchase and use electric vehicles, hybrid vehicles, and other clean energy vehicles; and encouraging green travel. (b) Strengthen environmental management, enhance monitoring and implement early warning systems: Establish a complete urban PAH monitoring system as well as a PAH pollution early warning mechanism. Formulate strict emission standards, better supervise and inspect implementation of these standards, and ensure that enterprises and individuals comply with these standards. Strengthen regional collaborative governance: enhance information sharing, joint law enforcement, and emergency responses among regions to jointly address PAH pollution issues. (c) Enhance public environmental awareness through various channels, such as television, newspapers, the internet, and social media; strengthen publicity and education on the hazards of PAH pollution and reduction; and raise public awareness of PAH pollution issues. Encourage public participation, fully listen to public opinions and suggestions, and uphold the public's right to environmental information, participation, and supervision (Bai et al., 2023; Kunal et al., 2021; Wu et al., 2020, 2024).

A suggested implementation path is as follows: In the short term (5–10 years), the focus should be on replacing scattered coal combustion sources and upgrading industries to realize clean heating in pilot cities. In the medium term (10–20 years), the scale of green power generation from eco‐friendly sources should be expanded, and full electrification in the transportation sector should be achieved. In the long term (after 20 years), a complementary multienergy system of “wind, solar, storage and hydrogen” should be constructed to promote full decarbonization of the energy system in northern cities (PRC, 2024).

In the first two decades of the 21st century, China experienced extensive growth, which has resulted in severe challenges to the environment. Therefore, the levels of PAH pollution in PM are becoming increasingly severe in Chinese urban areas, seriously affecting sustainable development and the health of city residents. In this study, 6,695 individual samples from 89 cities across China were reviewed, and the ecological and health risks of PAHs in PM in representative cities in China were also evaluated. However, due to the limited period analyzed and limitations in terms of the number of cities and samples, this study focused only on the harm resulting from exposure to atmospheric pollutants in densely populated cities across China. Other regions [including countries and continents (such as Asia, Europe, and the Americas)] should also coordinate control of atmospheric pollutants (Arushi et al., 2023; Bai et al., 2023; Sobhanardakani, 2018; Sobhanardakani et al., 2018).

## 4 Conclusions

In this comprehensive review, various data from published works were combined to examine the pollution levels, spatial distributions, ecological risks, and health risks of PAHs in China. PAH pollution levels varied greatly among Chinese cities. In general, the PAH content in PM indicated relatively severe PAH pollution and severe ecological risk in NC, while CC and SC had a higher population density and GDP. The mean ILCR and HI for adults in NC and the whole country were greater than 1.0E−6 and 10.0, respectively, indicating high potential health risks. According to accurate health risk assessments in China, the health risks due to PAHs in PM are much greater for adults than for juveniles. Therefore, adults need better protection against the risks (HI > 10.0, ILCR > 1.0E−6) posed by PAHs in PM, and the government should also address this issue. Producing and using new energy (solar energy, wind energy, etc.) may be a fundamental way to reduce environmental pollution in China and other developing countries. This research approach and the conclusions can be referenced or emulated by other developing countries.

## Conflict of Interest

The authors declare no conflicts of interest relevant to this study.

## Supporting information

Supporting Information S1

## Data Availability

The data on which this article is based are available in Dryad at (Wu et al., 2025).
