# Supplementary material for: Ecological and Health Risks of Polycyclic Aromatic Hydrocarbons in Particulate Matter in Chinese Cities
Source: Geohealth. 2025 Jun 6;9(6):e2024GH001126. doi: 10.1029/2024GH001126 (PMC12143031; doi:10.1029/2024GH001126)
Supplement: Supplementary file 1 — Supporting Information S1 [file GH2-9-e2024GH001126-s001.docx]

*Geohealth*

Supporting Information for

**Ecological and health risks of polycyclic aromatic hydrocarbons in particulate matter in Chinese cities**

Yongfu Wu^1,2,⁎^, Yuan Meng^1^, Han Zhang^2^, Lianglu Hao^2^, Tao Zeng^1^, Yan Shi^1^, Yunhe Chen^3^, Ni Qiao^4^, Yibin Ren^1^

^1^Affiliation for author 1aCollege of Agriculture and Biological Engineering, Longdong University, China

^2^College of Resources and Environmental Science, Gansu Agricultural University, China

^3^Qingyang Xifeng District People's Hospital, China

^4^Qingyang Ecological Environment Bureau, China

^⁎^Corresponding author: Yongfu Wu ([yongfu2006@126.com)](mailto:email@address.edu))

**Contents**

[Table S1 PAH content, population, and GDP in Chinese cities. 3](#_Toc32237)

[Table S2 Evaluation parameters for assessing the health risk of PAHs. 8](#_Toc25333)

[Table S3 Concentrations (ng/m](#_Toc9682)^[3](#_Toc9682)^[) of 16 PAHs. 9](#_Toc9682)

[Table S4 Health risk parameters of the four age groups in China 14](#_Toc23054)

[Table S5 Ratios between PAH concentration, population, and GDP 17](#_Toc3796)

[Table S6 Pearson correlations between PAH content in urban PM and geographical latitude, GDP and population. 18](#_Toc25661)

[Table S7 PAH ecological risk. 19](#_Toc4161)

[Table S8 Concentrations (ng/m](#_Toc12584)^[3](#_Toc12584)^[) and proportions (%) of PAHs. 25](#_Toc12584)

[Table S9 HIs of PAHs in Chinese urban PM. 30](#_Toc8840)

[Table S10 ILCRs of PAHs in Chinese urban PM. 35](#_Toc18782)

**Introduction**

Supplementary information with a table containing the location of urban, sample date, samples sites, samples number, range and mean of PAHs, population and GDP for each urban.

A number of studies have reported the contents of PAHs in PM in cities. These studies were published from 2000 to 2019 and include analyses of PAH contamination in response to rapid socioeconomic development in China, considering population increases, growing energy demand, and increases in industrial production. Publications in the scientific literature were selected following the eligibility criteria described below. Original studies published in English databases [e.g., Wiley Online Library (onlinelibrary.wiley.com/), [Elsevier](http://www.baidu.com/link?url=CA0484zbHoAiY8Cpmcjl0m1ft8CgSgsdvgbGh0FnGh_qQ1bN6UZkf0boREdDiZ3r) (https://www.sciencedirect.com/), and Springer Link (link.springer.com/)] and Chinese databases [Cqvip (www. cqvip. com/), Wanfang Data (www. wanfa ngdata. com. cn/ index. html), and China National Knowledge Infrastructure (www. cnki. net/)]. We searched for papers and books with the keywords “polycyclic aromatic hydrocarbon in the particulate matter”, “PAH in the particulate matter”, “polycyclic aromatic hydrocarbon in PM”, “PAH in PM”, and “City, China”. Thus, we retrieved almost all the relevant reports about PAHs in PM in Chinese cities. After removing duplicate or incomplete data, we collected data on the contents of 16 PAHs in PM in 89 cities in 34 provinces of China (Table S1). Based on their meteorological and geographical conditions and latitude, these cities were divided into three regions: NC, including 16 provincial administrative regions: Heilongjiang, Jilin, Liaoning, Anhui, Beijing, Hebei, Henan, Inner Mongolia, Shandong, Shanxi, Tianjin, Gansu, Shaanxi, Ningxia, Qinghai, and Xinjiang; CC, including 11 provincial administrative regions: Hubei, Hunan, Jiangsu, Jiangxi, Shanghai, Zhejiang, Yunnan, Chongqing, Sichuan, Xizang, and Guizhou; and SC, including 7 provincial administrative regions: Fujian, Guangxi, Guangdong, Hongkong, Macao, Taiwan, and Hainan (Table S1).

**Table S1** PAH content, population, and GDP in Chinese cities.

| Location | Date range | Sites | Number | PAH (ng/m^3^) | | Population  (million) | GDP  (billion yuan) | Reference |
| --- | --- | --- | --- | --- | --- | --- | --- | --- |
|  |  |  |  | Range | Mean |  |  |  |
| Harbin | 2013.1-2014.12 | 2 | 336 | 22.4-24.7 | 23.9 | 10.8 | 524.9 | (Fu et al., 2016) |
| Jiamusi | 2016.3-2016.12 | 2 | 71 | 0.4-279.4 | 32.8 | 2.3 | 76.3 | (Sun, 2018) |
| Changchun | 2011.12 | 8 | 24 | 41.8-74.3 | 55.4 | 7.5 | 590.4 | (Zhang, 2012) |
| Jilin | 2005.2-2005.9 | 4 | 12 | 141.5-3100.1 | 1201.4 | 4.1 | 141.7 | (Zhang, 2012) |
| Siping | 2005.2-2005.9 | 2 | 6 | 247.4-502.9 | 382.0 | 3.2 | 79.6 | (Zhang, 2012) |
| Tonghua | 2005.2-2005.9 | 2 | 6 | 303.3-1202.2 | 599.8 | 2.2 | 72.6 | (Zhang, 2012) |
| Baicheng | 2005.2-2005.9 | 2 | 6 | 66.6-649.9 | 342.5 | 1.9 | 49.2 | (Zhang, 2012) |
| Shenyang | 2004.4-2005.1 | 2 | 104 | 170.5-1053.8 | 603.9 | 8.3 | 647.0 | (Kong et al., 2010) |
| Dalian | 2004.4-2005.1 | 1 | 50 | # | 75.3 | 6.0 | 700.2 | (Kong et al., 2010) |
| Jinzhou | 2004.4-2005.1 | 2 | 101 | 107.9-191.5 | 150.2 | 2.9 | 107.3 | (Kong et al., 2010) |
| Fushun | 2004.4-2005.1 | 2 | 106 | 262.5-1900.9 | 1081.1 | 2.1 | 84.7 | (Kong et al., 2010) |
| Anshan | 2004.4-2005.1 | 3 | 147 | 411.2-923.0 | 602.6 | 3.4 | 174.5 | (Kong et al., 2010) |
| Panjin | 2012.1-2012.10 | 3 | 68 | # | 101.3 | 1.4 | 128.1 | (Tao, 2015) |
| Beijing | 2016.1-2016.12 | 1 | 218 | 12.5-156.0 | 66.4 | 21.5 | 3537.1 | (Chao et al., 2019) |
| Shijiazhuang | 2016.11-2017.11 | 1 | ≥1 | 25.1-35.0 | 32.7 | 10.4 | 581.0 | (An et al., 2018) |
| Zhangjiakou | 2016.11-2017.10 | 1 | ≥1 | 8.7-316.1 | 48.9 | 4.4 | 155.1 | (An et al., 2018) |
| Baoding | 2016.11-2017.11 | 1 | ≥1 | 29.3-229.8 | 66.0 | 9.4 | 322.4 | (An et al., 2018) |
| Tangshan | 2016.11-2017.10 | 1 | ≥1 | 12.6-115.8 | 35.9 | 8.0 | 689.0 | (An et al., 2018) |
| Handan | 2012.10-2013.8 | 3 | 144 | 3.7-136.5 | 40.9 | 9.6 | 348.6 | (Ling, 2014) |
| Hengshui | 2015.1-2015.2 | 1 | 30 | # | 169.0 | 4.5 | 150.5 | (Niu et al., 2017) |
| Zhengzhou | 2011.3-2014.1 | 1 | 188 | 7.0-961.0 | 177.3 | 10.4 | 1159.0 | (Wang et al., 2015b) |
| Xinxiang | 2013.10-2014.8 | ≥1 | 12 | 10.0-219.4 | 81.9 | 5.8 | 291.8 | (Liu et al., 2017) |
| Hohhot | 2015.1-2015.12 | 2 | 130 | 23.3-41.0 | 32.5 | 3.1 | 279.2 | (Wei et al., 2017) |
| Baotou | 2012.9-2013.5 | 4 | 36 | 17.8-802.8 | 230.2 | 2.9 | 271.5 | (Lu, 2015) |
| Ordos | 2005.9 | 1 | 5 | # | 26.6 | 0.6 | 70.2 | (Wu et al., 2014) |
| Jinan | 2014.1-2017.12 | 2 | 738 | 16.3-89.9 | 45.3 | 8.9 | 944.3 | (Yu et al., 2018) |
| Qingdao | 2013.12-2017.8 | 1 | 12 | # | 30.3 | 9.5 | 1174.1 | (Wang, 2015b) |
| Taiyuan | 2012.3-2013.1 | 1 | 62 | 10.4-215.9 | 88.1 | 4.5 | 402.9 | (Zhang et al., 2016) |
| Jinzhong | 2013.12-2017.8 | 1 | 12 | # | 910.5 | 3.4 | 146.0 | (Wang, 2015b) |
| Tianjin | 2005.7-2005.8 | 4 | 10 | 867.5 | 865.8 | 15.6 | 1410.4 | (Hu et al., 2007) |
| Lanzhou | 2015.1-2016.12 | 2 | 342 | 3.5-6.6 | 5.5 | 3.3 | 283.7 | (Song et al., 2018) |
| Jinchang | 2003.1-2003.7 | 1 | 4 | 4.7-213.0 | 85.8 | 0.5 | 34.0 | (Wang et al., 2006) |
| Xi'an | 2016.12-2017.11 | 2 | 68 | 14.3-266.0 | 63.3 | 10.2 | 932.1 | (Wang et al., 2019) |
| Yulin | 2003.1-2003.7 | 1 | 4 | # | 94.6 | 3.4 | 413.6 | (Wang et al., 2006) |
| Yinchuan | 2015.1-2016.10 | 1 | 56 | 32.9-250.9 | 98.1 | 2.3 | 21.6 | (Tian et al., 2017) |
| Xining | 2009.9-2010.5 | 7 | 136 | 37.5-142.9 | 68.8 | 2.4 | 61.9 | (Shi et al., 2012) |
| Urumqi | 2012.1-2012.12 | 1 | 44 | 682.2-968.7 | 802.7 | 3.6 | 341.3 | (Kermilla, 2014) |
| Hotan | 2014.1-2014.11 | 1 | 117 | 2.2-429.5 | 93.4 | 2.5 | 30.6 | (Geng et al., 2018) |
| Changji | 2004.11-2005.4 | 2 | 18 | 12.2-188.9 | 72.0 | 1.6 | 132.5 | (Diao, 2006) |
| Karamay | 2015.9-2015.12 | 1 | 33 | 0.8-103.6 | 31.4 | 0.5 | 88.7 | (Turap et al., 2018) |
| Shihezi | 2008.8-2009.7 | ≥1 | 52 | 41.4-1130.0 | 275.9 | 3.2 | 274.7 | (Ma et al., 2018) |
| NC Mean |  |  |  |  | 241.3 | 5.4 | 437.2 |  |
| Hefei | 2015.8-2016.1 | 1 | 120 | 5.9-63.4 | 40.6 | 7.7 | 940.9 | (Hu et al., 2018a) |
| Huainan | 2014.3-2015.1 | 6 | 24 | 16.9-79.6 | 31.1 | 3.9 | 129.6 | (Hu, 2016) |
| Tongling | 2014.8-2015.1 | 3 | 35 | 31.2-76.4 | 54.9 | 1.7 | 96.0 | (Zhang et al., 2018) |
| Wuhan | 2014.3-2015.1 | 1 | 115 | 11.3-48.2 | 25.7 | 11.2 | 1622.3 | (Zhang et al., 2019) |
| Yichang | 2015.7-2016.6 | 2 | ≥84 | 0-79.8 | 19.9 | 4.1 | 446.1 | (Yang et al., 2017a) |
| Huangshi | 2013 | 1 | 60 | 1.5-44.5 | 10.8 | 2.5 | 176.7 | (Hu et al., 2018b) |
| Changsha | 2008.5-2008.9 | 3 | 23 | 15.5-32.5 | 21.8 | 8.4 | 1157.4 | (Yang et al., 2010) |
| Xiangtan | 2015.12-2016.2 | 3 | 57 | 13.6-29.4 | 19.2 | 2.9 | 225.8 | (Wang et al., 2016) |
| Nanjing | 2014.1-2014.11 | 1 | 63 | 24.5-30.0 | 28.1 | 8.5 | 1403.0 | (Li, 2016) |
| Changzhou | 2016.1-2016.8 | 1 | 55 | 2.8-128.2 | 31.8 | 4.7 | 740.1 | (Gu et al., 2017) |
| Xuzhou | # | 3 | 42 | 18.6-388.0 | 164.7 | 8.8 | 715.1 | (Chen et al., 2013a) |
| Wuxi | 2016.1-2016.12 | 1 | 88 | # | 16.5 | 5.0 | 1185.2 | (Wang et al., 2018) |
| Zhenjiang | 2016.1-2016.12 | 1 | 86 | # | 12.4 | 3.2 | 412.7 | (Wang et al., 2018) |
| Nantong | 2016.6-2017.5 | 1 | 84 | 7.4-20.4 | 12.9 | 7.3 | 938.3 | (Guo et al., 2018) |
| Nanchang | 2015.7-2016.1 | 1 | ≥1 | 7.5-63.6 | 35.4 | 5.4 | 559.6 | (He et al., 2018) |
| Jiujiang | 2015.7-2016.1 | 1 | ≥1 | 6.2-30.6 | 18.5 | 4.9 | 312.1 | (He et al., 2018) |
| Shanghai | 2014.12-2015.11 | 1 | 122 | 6.8-61.9 | 33.2 | 24.3 | 3815.5 | (Liu et al., 2018) |
| Hangzhou | 2015.3-2016.2 | 1 | 84 | 4.5-13.6 | 9.1 | 10.4 | 1537.3 | (Lu et al., 2017) |
| Huzhou | 2013.7-2014.3 | 4 | 144 | 4.8-24.0 | 11.5 | 2.7 | 312.2 | (Fei et al., 2015) |
| Ningbo | 2009.7-2010.3 | 1 | 36 | 0.4-52.8 | 12.9 | 6.1 | 1198.5 | (Liu et al., 2014) |
| Wenzhou | 2015.1-2015.10 | 4 | 112 | 5.1-81.6 | 32.4 | 9.3 | 660.6 | (Zheng et al., 2017) |
| Taizhou | 2015.7-2016.3 | 6 | 168 | 7.5-33.4 | 20.9 | 6.1 | 513.4 | (Tao et al., 2017) |
| Jinhua | 2016.1-2016.12 | 1 | 84 | 2.1-46.5 | 8.7 | 4.9 | 456.0 | (He et al., 2017) |
| Kunming | 2013.4-2014.1 | 3 | 20 | # | 28.3 | 7.0 | 647.6 | (Bi et al., 2015) |
| Yuxi | 2014.8-2015.7 | 6 | 120 | 0.4-80.8 | 13.4 | 2.4 | 195.0 | (Huang, 2015) |
| Chongqing | 2012.4 | 4 | 20 | 31.7-189.3 | 107.1 | 31.2 | 2360.6 | (Chen et al., 2013b) |
| Chengdu | 2013.10-2014.8 | ≥1 | 15 | 3.8-44.9 | 18.8 | 16.6 | 1701.3 | (Liu et al., 2017) |
| Mianyang | 2013.12-2017.8 | 1 | 12 | # | 22.0 | 4.9 | 285.6 | (Wang, 2015b) |
| Lhasa | 2008.8-2009.7 | ≥1 | 50 | 66.3-937.0 | 75.5 | 0.6 | 61.8 | (Ma et al., 2018) |
| Guiyang | 2012.9-2013.8 | 3 | 144 | 35.0-43.0 | 38.3 | 5.0 | 404.0 | (Fan et al., 2019) |
| CC Mean |  |  |  |  | 33.0 | 7.4 | 840.3 |  |
| Xiamen | 2013.1-2013.2 | 1 | 6-8 | # | 4.4 | 4.3 | 599.5 | (Leung et al., 2014) |
| Longyan | 2003.12-2004.3 | 3 | 28 | 230.1-479.4 | 359.2 | 2.6 | 267.9 | (He et al., 2008) |
| Nanning | 2011.8-2012.1 | 2 | ≥4 | 35.3-121.5 | 64.0 | 7.3 | 450.7 | (Miao et al., 2016) |
| Baize | 2012.11-2013.7 | 1 | 120 | 6.9-142.3 | 33.2 | 3.7 | 125.8 | (Shi et al., 2015) |
| Liuzhou | 2002.12-2003.3 | 4 | 48 | 57.6-190.2 | 102.8 | 4.1 | 312.8 | (He et al., 2003) |
| Guangzhou | 2013.7-2014.5 | 4 | 383 | 0.3-57.0 | 9.8 | 15.3 | 2362.9 | (Yu et al., 2016) |
| Shenzhen | 2012.9-2013.8 | 1 | 84 | 2.8-85.0 | 18.4 | 13.4 | 2692.7 | (Sun et al., 2015) |
| Qingyuan | 2009.8-2010.2 | 2 | 80 | 5.4-107.5 | 48.1 | 3.9 | 169.8 | (Wei et al., 2012) |
| Shaoguan | 2015.1-2015.10 | 5 | ≥80 | 99.1-118.9 | 102.4 | 3.0 | 131.8 | (Luo, 2017) |
| Shantou | 2004.8-2004.9 | 1 | 30 | 22.7-263.0 | 102.6 | 5.6 | 269.4 | (Deng et al., 2006) |
| Dongguan | 2011.6 | 4 | 28 | 12.6-194.0 | 84.2 | 2.5 | 948.3 | (Yang et al., 2012) |
| Hongkong | 2014.2 | 2 | 7 | 1.4-3.1 | 2.5 | 7.5 | 2547.6 | (Fan et al., 2017) |
| Macao | 2010.1-2010.5 | 2 | 18 | 0.8-4.2 | 2.8 | 0.7 | 378.9 | (Zheng et al., 2011) |
| Taipei | 2014.6-2014.12 | 2 | 41 | 1.3-3.5 | 2.5 | 2.5 | 617.0 | (Hsu et al., 2019) |
| Taichung | 2014.6-2015.1 | 2 | 48 | 2.2-2.3 | 2.2 | 2.8 | 487.6 | (Zhu et al., 2019) |
| Miaoli | 2010.1-2010.8 | 20 | 35 | 12.0-40.3 | 26.2 | 0.6 | 84.5 | (Wu et al., 2012) |
| Hsinchu | 2014.9-2015.8 | 1 | 37 | 0.4-4.0 | 1.6 | 0.6 | 96.3 | (Yang et al., 2017b) |
| Haikou | 2014.11-2015.1 | 2 | 37 | 4.1-5.3 | 4.9 | 2.3 | 167.2 | (Liu et al., 2016) |
| SC Mean |  |  |  |  | 54.0 | 4.6 | 706.2 |  |

GDP: gross domestic product in urban areas; population: permanent urban population; TSP: total suspended particulate matter.

**Table S2** Evaluation parameters for assessing the health risk of PAHs.

| PAH | *LOD* | *TEF* | *RfC_i_*  (mg/kg/d) | *CSF_i_*  (kg d/mg) |
| --- | --- | --- | --- | --- |
| Nap (naphthalene) | 0.009 | 0.001 | 8.57E-4 |  |
| Acy (acenaphthylene) | 0.002 | 0.001 | 3.00E-2 |  |
| Ace (acenaphthene) | 0.002 | 0.001 | 3.00E-2 |  |
| Flu (fluorene) | 0.007 | 0.001 | 2.00E-2 |  |
| Ant (anthracene) | 0.003 | 0.01 | 1.50E-2 |  |
| Phe (phenanthrene) | 0.015 | 0.001 | 1.50E-1 |  |
| Fla (fluoranthene) | 0.006 | 0.001 | 2.00E-2 |  |
| Pyr (pyrene) | 0.002 | 0.001 | 1.50E-2 |  |
| BP (benzo[g,h,i]perylene) | 0.002 | 0.01 | 1.50E-2 |  |
| BaA (benzo[a]anthracene) | 0.002 | 0.1 |  | 3.10E-1 |
| Chr (chrysene) | 0.002 | 0.01 |  | 3.10E-3 |
| BbF (benzo[b]fluoranthene) | 0.002 | 0.1 |  | 3.10E-1 |
| BkF (benzo[k]fluoranthene) | 0.002 | 0.1 |  | 3.10E-2 |
| BaP (benzo[a]pyrene) | 0.002 | 1.0 |  | 3.10 |
| DA (dibenz[a,h]anthracene) | 0.002 | 0.001 |  | 3.10 |
| IP (indeno[1,2,3-c,d] pyrene) | 0.002 | 0.1 |  | 3.10E-1 |

*LOD*: limit of detection; TEF: toxic equivalency factor; *RfC_i_*: reference content for inhalation (USEPA, 2005; USEPA, 2010; USEPA, 2017).

**Table S3** Concentrations (ng/m^3^) of 16 PAHs.

| City | Nap | Acy | Ace | Flu | Ant | Phe | Fla | Pyr | BaA | Chr | BbF | BkF | BaP | DA | BP | IP | *TEQ* |
| --- | --- | --- | --- | --- | --- | --- | --- | --- | --- | --- | --- | --- | --- | --- | --- | --- | --- |
| Harbin | 1.4 | 0.7 | 0.2 | 0.3 | 0.9 | 4.2 | 4.5 | 4.1 | 2.9 | 0.8 | 0.9 | 0.5 | 0.3 | 0.5 | 0.4 | 1.3 | *1.3* |
| Jiamusi | 0.0 | 0.1 | 0.0 | 0.0 | 0.2 | 0.8 | 3.9 | 4.5 | 4.4 | 5.2 | 2.7 | 3.1 | 3.3 | 0.6 | 1.9 | 2.1 | *5.2* |
| Changchun | 0.0 | 0.0 | 0.4 | 5.6 | 4.0 | 5.7 | 0.0 | 18.7 | 3.8 | 1.0 | 4.0 | 2.3 | 1.3 | 1.1 | 3.9 | 3.6 | *3.9* |
| Jilin | 1.2 | 0.0 | 0.9 | 1.6 | 3.8 | 20.2 | 108.0 | 86.6 | 152.0 | 263.0 | 230.0 | 149.0 | 61.6 | 11.9 | 42.0 | 69.6 | *136.9* |
| Siping | 1.6 | 0.0 | 1.1 | 1.0 | 2.4 | 15.6 | 43.2 | 38.1 | 49.0 | 62.5 | 60.0 | 37.9 | 22.3 | 2.7 | 20.9 | 23.9 | *43.0* |
| Tonghua | 1.1 | 0.0 | 0.8 | 1.0 | 3.2 | 21.1 | 63.1 | 57.6 | 72.4 | 100.0 | 109.0 | 68.9 | 41.4 | 3.3 | 37.2 | 19.0 | *73.2* |
| Baicheng | 0.6 | 0.0 | 0.4 | 0.6 | 1.8 | 10.9 | 40.6 | 33.4 | 37.6 | 49.9 | 63.0 | 39.8 | 22.1 | 0.7 | 17.7 | 23.4 | *39.9* |
| Shenyang | 4.1 | 0.0 | 3.8 | 0.0 | 32.3 | 81.1 | 163.0 | 142.0 | 34.4 | 43.9 | 39.5 | 9.8 | 18.6 | 16.3 | 3.1 | 12.0 | *45.7* |
| Dalian | 1.5 | 0.0 | 1.1 | 0.0 | 1.0 | 7.1 | 12.5 | 8.1 | 6.1 | 7.6 | 8.9 | 2.9 | 5.4 | 6.7 | 1.6 | 4.8 | *14.4* |
| Jinzhou | 0.8 | 0.0 | 1.5 | 0.0 | 2.6 | 8.2 | 36.2 | 22.5 | 15.6 | 9.8 | 15.2 | 4.9 | 10.1 | 12.2 | 2.1 | 8.5 | *26.9* |
| Fushun | 1.1 | 0.0 | 2.1 | 0.0 | 9.6 | 47.6 | 315.0 | 318.0 | 104.0 | 113.0 | 69.6 | 19.9 | 29.6 | 27.4 | 6.5 | 17.7 | *80.1* |
| Anshan | 1.0 | 0.0 | 147.0 | 0.0 | 6.6 | 19.4 | 76.1 | 72.7 | 32.0 | 32.3 | 36.5 | 28.1 | 29.9 | 65.1 | 37.1 | 18.8 | *107.6* |
| Panjing | 0.6 | 0.0 | 1.1 | 0.0 | 1.8 | 5.5 | 24.2 | 15.0 | 10.5 | 6.6 | 10.2 | 3.4 | 6.8 | 8.2 | 1.7 | 5.7 | *18.2* |
| Beijing | 0.3 | 0.1 | 0.5 | 0.5 | 0.6 | 3.7 | 6.0 | 5.0 | 4.2 | 5.7 | 14.1 | 4.4 | 8.2 | 5.7 | 6.3 | 1.1 | *16.4* |
| Shijiazhuang | 0.0 | 0.1 | 0.0 | 0.0 | 2.0 | 0.1 | 2.2 | 1.3 | 1.6 | 4.3 | 4.5 | 3.9 | 6.6 | 2.2 | 2.2 | 1.7 | *10.1* |
| Zhangjiakou | 0.3 | 0.3 | 0.1 | 0.3 | 0.5 | 1.6 | 4.6 | 6.7 | 5.3 | 7.4 | 7.1 | 2.1 | 4.8 | 1.4 | 2.9 | 3.5 | *8.1* |
| Baoding | 2.1 | 3.6 | 3.1 | 4.8 | 1.8 | 4.9 | 4.5 | 3.0 | 7.5 | 3.8 | 4.5 | 6.1 | 3.9 | 3.3 | 3.9 | 5.2 | *9.6* |
| Tangshan | 0.0 | 0.0 | 0.0 | 0.0 | 0.0 | 0.1 | 3.9 | 3.0 | 2.6 | 3.5 | 4.3 | 1.6 | 3.6 | 3.7 | 6.0 | 3.6 | *8.6* |
| Handan | 0.0 | 0.0 | 0.0 | 0.0 | 1.5 | 4.4 | 6.4 | 4.9 | 2.0 | 4.6 | 7.4 | 0.9 | 3.4 | 0.0 | 2.9 | 2.5 | *4.8* |
| Hengshui | 4.0 | 0.9 | 1.8 | 5.7 | 1.8 | 7.4 | 12.6 | 12.2 | 31.1 | 21.1 | 17.4 | 10.4 | 15.4 | 1.0 | 10.3 | 15.9 | *24.3* |
| Zhengzhou | 3.1 | 1.1 | 1.7 | 1.4 | 7.7 | 7.0 | 19.1 | 15.1 | 15.5 | 20.4 | 29.6 | 17.0 | 10.1 | 5.1 | 11.0 | 12.4 | *23.1* |
| Xinxiang | 0.1 | 0.0 | 0.0 | 0.3 | 1.7 | 3.5 | 12.4 | 10.6 | 8.9 | 12.8 | 10.1 | 7.3 | 4.5 | 0.5 | 4.3 | 4.9 | *8.3* |
| Hohhot | 12.2 | 1.1 | 1.0 | 0.2 | 0.3 | 0.5 | 0.2 | 2.1 | 1.9 | 2.3 | 3.8 | 1.0 | 1.9 | 3.4 | 0.5 | 0.1 | *6.0* |
| Baotou | 1.2 | 0.6 | 0.3 | 1.3 | 2.2 | 10.0 | 24.4 | 22.3 | 36.7 | 33.0 | 27.5 | 12.6 | 19.8 | 3.6 | 15.4 | 19.3 | *33.6* |
| Ordos | 0.3 | 0.0 | 0.0 | 0.1 | 0.1 | 0.7 | 2.4 | 2.1 | 1.8 | 3.1 | 2.9 | 2.1 | 1.8 | 0.7 | 6.1 | 2.4 | *3.5* |
| Jinan | 1.1 | 1.3 | 0.1 | 0.2 | 0.5 | 1.2 | 4.2 | 3.0 | 3.2 | 6.7 | 9.1 | 1.7 | 3.1 | 0.8 | 4.7 | 4.4 | *5.8* |
| Qingdao | 0.2 | 0.0 | 0.1 | 0.3 | 0.2 | 2.8 | 5.8 | 3.9 | 1.6 | 5.2 | 2.2 | 3.5 | 1.7 | 0.2 | 1.4 | 1.2 | *2.8* |
| Taiyuan | 0.0 | 0.0 | 0.4 | 0.0 | 1.7 | 9.4 | 17.8 | 20.7 | 7.0 | 7.4 | 1.2 | 2.4 | 6.5 | 0.7 | 7.3 | 5.6 | *8.9* |
| Jinzhong | 1.3 | 0.7 | 1.3 | 4.5 | 11.9 | 73.1 | 193.0 | 121.0 | 58.7 | 148.0 | 80.7 | 91.7 | 55.4 | 9.4 | 36.3 | 23.5 | *92.6* |
| Tianjin | 0.0 | 0.0 | 0.0 | 19.5 | 56.0 | 145.8 | 84.9 | 47.3 | 35.4 | 53.9 | 34.4 | 34.8 | 26.2 | 13.3 | 7.3 | 307.0 | *82.1* |
| Lanzhou | 0.7 | 0.0 | 0.1 | 0.4 | 1.1 | 0.1 | 0.5 | 0.5 | 0.3 | 0.4 | 0.5 | 0.3 | 0.2 | 0.1 | 0.1 | 0.2 | *0.4* |
| Jinchang | 0.0 | 0.0 | 0.0 | 3.0 | 0.0 | 1.8 | 3.0 | 3.7 | 5.5 | 8.0 | 31.6 | 4.6 | 4.8 | 3.1 | 8.4 | 8.3 | *13.0* |
| Xi'an | 1.5 | 3.3 | 2.3 | 1.8 | 3.0 | 3.1 | 6.7 | 3.0 | 9.9 | 3.2 | 9.3 | 1.9 | 3.8 | 3.8 | 3.9 | 2.8 | *10.1* |
| Yulin | 0.0 | 0.0 | 0.0 | 7.4 | 0.0 | 2.1 | 7.4 | 7.4 | 7.5 | 5.0 | 28.5 | 5.1 | 6.5 | 1.1 | 7.0 | 9.6 | *12.7* |
| Yinchuan | 11.9 | 0.1 | 0.5 | 0.0 | 1.2 | 15.6 | 8.5 | 6.3 | 9.9 | 5.5 | 17.2 | 0.0 | 5.5 | 3.4 | 6.0 | 6.5 | *12.4* |
| Xining | 0.0 | 0.0 | 0.0 | 0.0 | 2.1 | 9.0 | 29.4 | 9.6 | 5.1 | 4.0 | 3.9 | 3.2 | 1.6 | 0.0 | 0.7 | 0.2 | *2.9* |
| Urumqi | 25.1 | 30.5 | 19.5 | 32.9 | 8.6 | 41.6 | 67.0 | 71.9 | 53.5 | 78.9 | 82.6 | 76.0 | 6.5 | 51.6 | 77.9 | 78.6 | *89.1* |
| Hotan | 2.6 | 0.3 | 1.7 | 0.7 | 1.4 | 3.2 | 13.3 | 11.7 | 8.2 | 7.4 | 10.4 | 7.8 | 6.5 | 3.1 | 6.1 | 9.0 | *13.3* |
| Changji | 6.4 | 0.2 | 0.1 | 0.7 | 1.8 | 5.1 | 9.3 | 6.9 | 5.2 | 5.8 | 7.1 | 7.3 | 4.2 | 1.4 | 5.3 | 5.2 | *8.2* |
| Karamay | 0.0 | 0.0 | 0.0 | 0.4 | 0.2 | 1.6 | 6.5 | 7.8 | 1.6 | 2.0 | 3.7 | 2.9 | 1.2 | 0.1 | 1.9 | 1.5 | *2.3* |
| Shihezi | 16.0 | 24.7 | 4.1 | 32.7 | 10.6 | 77.2 | 35.7 | 22.0 | 8.9 | 10.1 | 10.7 | 5.8 | 5.8 | 1.5 | 5.2 | 4.9 | *10.8* |
| **NC Mean** | **2.6** | **1.7** | **4.9** | **3.2** | **4.7** | **16.7** | **36.1** | **30.6** | **21.1** | **28.5** | **27.2** | **16.8** | **11.6** | **6.9** | **10.4** | **18.3** | ***27.3*** |
| KunMing | 0.3 | 0.9 | 0.0 | 0.0 | 0.2 | 2.1 | 2.1 | 3.1 | 1.9 | 3.4 | 3.5 | 1.7 | 3.0 | 0.1 | 3.1 | 2.9 | *4.2* |
| Yuxi | 0.3 | 0.6 | 0.3 | 0.7 | 0.0 | 0.4 | 1.4 | 1.6 | 0.7 | 1.0 | 1.4 | 0.7 | 1.1 | 0.1 | 1.6 | 1.5 | *1.6* |
| Chongqing | 0.6 | 0.5 | 0.0 | 0.7 | 0.7 | 0.9 | 2.7 | 1.7 | 6.0 | 10.3 | 23.2 | 17.1 | 8.3 | 6.9 | 12.2 | 15.3 | *21.6* |
| Chengdu | 0.1 | 0.0 | 0.0 | 0.1 | 0.2 | 0.6 | 1.9 | 1.9 | 2.0 | 2.1 | 4.1 | 0.9 | 1.8 | 0.2 | 1.3 | 1.6 | *2.9* |
| Mianyang | 0.1 | 0.1 | 0.0 | 0.5 | 0.1 | 1.1 | 2.2 | 1.6 | 1.1 | 3.9 | 1.5 | 3.7 | 2.1 | 0.3 | 1.9 | 1.8 | *3.2* |
| Lhasa | 7.9 | 2.4 | 0.7 | 9.5 | 2.2 | 29.1 | 7.8 | 6.0 | 1.2 | 1.9 | 1.9 | 1.3 | 1.1 | 0.2 | 1.2 | 1.1 | *2.0* |
| Guiyang | 1.8 | 5.3 | 6.1 | 0.8 | 1.4 | 5.8 | 0.8 | 1.3 | 1.6 | 1.2 | 2.8 | 1.2 | 1.4 | 2.0 | 3.1 | 1.7 | *4.2* |
| Hefei | 0.1 | 0.0 | 0.0 | 0.0 | 0.6 | 0.6 | 0.8 | 1.2 | 0.5 | 3.2 | 0.9 | 0.9 | 1.4 | 6.8 | 16.6 | 7.0 | *9.4* |
| Huainan | 0.3 | 1.0 | 0.7 | 1.2 | 0.7 | 1.4 | 2.3 | 2.6 | 3.9 | 3.3 | 2.9 | 2.4 | 1.0 | 1.8 | 3.2 | 2.4 | *4.0* |
| Tongling | 0.8 | 1.0 | 1.2 | 1.3 | 2.8 | 2.1 | 4.8 | 4.0 | 4.8 | 4.6 | 4.6 | 4.6 | 4.5 | 4.1 | 4.7 | 5.0 | *10.6* |
| Wuhan | 1.0 | 0.1 | 0.0 | 0.1 | 0.2 | 2.8 | 2.1 | 2.2 | 1.5 | 3.1 | 4.9 | 1.4 | 1.9 | 0.2 | 2.0 | 2.2 | *3.2* |
| Yichang | 0.3 | 0.0 | 0.0 | 0.0 | 0.2 | 0.7 | 1.6 | 1.4 | 2.2 | 1.7 | 3.4 | 1.3 | 1.8 | 0.2 | 2.2 | 2.9 | *3.0* |
| Huangshi | 0.5 | 0.1 | 0.0 | 0.0 | 0.0 | 0.8 | 1.8 | 1.2 | 0.8 | 0.8 | 0.7 | 0.5 | 0.5 | 0.5 | 1.1 | 1.5 | *1.4* |
| Changsha | 0.2 | 2.4 | 0.1 | 1.5 | 1.5 | 1.0 | 2.2 | 1.6 | 0.7 | 1.3 | 1.4 | 1.5 | 1.7 | 1.0 | 1.9 | 1.8 | *3.2* |
| Xiangtan | 0.0 | 0.0 | 0.3 | 1.2 | 0.8 | 2.9 | 1.6 | 0.9 | 1.0 | 1.7 | 2.3 | 1.7 | 1.7 | 0.5 | 1.5 | 1.1 | *2.8* |
| Nanjing | 0.3 | 0.1 | 0.1 | 0.2 | 0.2 | 1.1 | 2.5 | 1.9 | 1.8 | 2.7 | 5.8 | 1.3 | 2.1 | 0.9 | 3.5 | 3.6 | *4.4* |
| Changzhou | 2.5 | 0.1 | 0.1 | 0.6 | 0.2 | 1.5 | 2.0 | 2.1 | 2.4 | 3.7 | 3.1 | 1.4 | 2.7 | 0.5 | 4.1 | 4.8 | *4.5* |
| Xuzhou | 5.1 | 5.0 | 2.4 | 2.2 | 7.5 | 7.4 | 24.7 | 22.9 | 8.0 | 23.3 | 3.5 | 4.8 | 10.8 | 10.3 | 12.1 | 14.7 | *24.7* |
| Wuxi | 0.3 | 0.1 | 0.3 | 0.1 | 0.3 | 0.7 | 1.0 | 0.9 | 1.6 | 0.9 | 0.7 | 4.0 | 1.3 | 0.6 | 3.2 | 0.5 | *2.7* |
| Zhenjiang | 0.3 | 0.3 | 0.3 | 0.0 | 0.0 | 0.7 | 1.1 | 1.2 | 0.6 | 1.0 | 2.1 | 0.7 | 0.9 | 0.5 | 1.3 | 1.4 | *1.9* |
| Nantong | 0.0 | 0.0 | 0.0 | 0.0 | 0.0 | 0.5 | 1.5 | 1.2 | 1.0 | 1.5 | 2.2 | 0.7 | 1.7 | 0.3 | 2.3 | 0.0 | *2.4* |
| Nanchang | 2.1 | 0.0 | 0.8 | 1.9 | 0.2 | 3.0 | 0.8 | 0.9 | 11.9 | 1.4 | 4.6 | 1.9 | 2.8 | 0.0 | 2.9 | 0.2 | *4.7* |
| Jiujiang | 0.0 | 0.0 | 0.7 | 1.5 | 0.4 | 1.3 | 0.8 | 0.3 | 4.5 | 0.2 | 1.6 | 1.3 | 1.5 | 0.0 | 3.0 | 1.4 | *2.4* |
| Shanghai | 1.9 | 1.0 | 0.2 | 0.6 | 1.0 | 3.1 | 3.9 | 2.8 | 3.3 | 2.4 | 1.9 | 1.9 | 3.0 | 0.4 | 2.5 | 3.3 | *4.5* |
| Hangzhou | 0.8 | 0.9 | 0.9 | 1.1 | 1.6 | 0.9 | 1.3 | 1.4 | 1.8 | 1.8 | 1.9 | 2.0 | 1.8 | 2.4 | 2.3 | 2.4 | *5.0* |
| Huzhou | 0.0 | 0.0 | 0.0 | 0.0 | 0.1 | 0.6 | 1.3 | 1.6 | 0.5 | 1.3 | 1.3 | 0.5 | 0.6 | 0.1 | 2.4 | 1.2 | *1.1* |
| Ningbo | 0.0 | 0.0 | 0.0 | 0.0 | 0.2 | 0.4 | 1.1 | 0.9 | 0.8 | 1.3 | 2.2 | 0.8 | 1.0 | 0.6 | 1.6 | 2.0 | *2.2* |
| Wenzhou | 0.5 | 1.3 | 0.9 | 1.0 | 0.6 | 4.0 | 3.1 | 2.1 | 3.7 | 2.4 | 3.2 | 1.0 | 1.9 | 0.3 | 2.3 | 4.1 | *3.4* |
| Taizhou | 0.2 | 0.1 | 0.1 | 0.2 | 0.2 | 0.9 | 1.0 | 0.9 | 1.2 | 1.9 | 2.9 | 2.8 | 2.0 | 1.0 | 3.5 | 2.0 | *4.0* |
| Jinhua | 0.1 | 0.1 | 0.1 | 0.1 | 0.1 | 0.2 | 0.2 | 0.4 | 0.5 | 0.7 | 2.6 | 0.5 | 0.8 | 0.3 | 0.6 | 1.4 | *1.6* |
| **CC Mean** | **0.9** | **0.8** | **0.5** | **0.9** | **0.8** | **2.6** | **2.7** | **2.5** | **2.5** | **3.0** | **3.3** | **2.2** | **2.3** | **1.4** | **3.5** | **3.1** | **4.9** |
| Xiamen | 0.0 | 0.0 | 0.0 | 0.0 | 0.1 | 0.0 | 0.3 | 0.2 | 0.6 | 0.5 | 0.4 | 0.4 | 0.4 | 0.4 | 0.5 | 0.6 | *1.0* |
| Longyan | 2.5 | 11.8 | 0.0 | 4.6 | 1.5 | 91.1 | 81.9 | 69.0 | 1.7 | 67.2 | 0.0 | 20.9 | 7.0 | 0.0 | 0.0 | 0.0 | *10.2* |
| Nanning | 14.5 | 0.4 | 0.9 | 8.1 | 0.7 | 17.9 | 5.8 | 9.3 | 1.1 | 2.5 | 1.2 | 0.5 | 0.2 | 0.0 | 0.4 | 0.5 | *0.6* |
| Baize | 1.9 | 0.0 | 0.4 | 0.6 | 0.4 | 1.7 | 2.0 | 2.2 | 2.2 | 3.7 | 3.8 | 3.5 | 2.0 | 1.5 | 3.8 | 3.5 | *4.8* |
| Liuzhou | 2.8 | 2.0 | 2.0 | 1.9 | 5.5 | 4.9 | 10.6 | 8.4 | 18.4 | 13.1 | 5.6 | 9.2 | 10.7 | 1.5 | 2.8 | 3.4 | *16.1* |
| Guangzhou | 0.0 | 0.0 | 0.0 | 0.2 | 0.1 | 0.4 | 0.7 | 0.7 | 0.5 | 0.9 | 1.3 | 1.3 | 0.8 | 0.2 | 1.2 | 1.5 | *1.4* |
| Shenzhen | 0.7 | 0.0 | 0.0 | 0.1 | 0.1 | 0.6 | 1.0 | 1.0 | 0.6 | 1.7 | 3.2 | 0.9 | 1.2 | 0.4 | 4.3 | 2.6 | *2.4* |
| Qingyuan | 0.0 | 0.0 | 0.0 | 0.1 | 0.1 | 0.9 | 2.4 | 2.0 | 0.9 | 1.9 | 5.5 | 2.3 | 3.4 | 2.2 | 15.2 | 11.2 | *7.7* |
| Shaoguan | 2.6 | 1.7 | 1.5 | 1.9 | 4.7 | 4.8 | 9.6 | 7.7 | 16.4 | 0.0 | 8.8 | 15.1 | 10.0 | 1.6 | 12.3 | 3.7 | *16.2* |
| Shantou | 3.3 | 0.0 | 0.1 | 0.0 | 0.5 | 2.5 | 2.8 | 6.8 | 2.8 | 8.5 | 11.8 | 11.8 | 8.9 | 1.5 | 19.4 | 21.9 | *15.5* |
| Dongguan | 0.0 | 3.3 | 1.1 | 41.0 | 0.1 | 0.0 | 25.9 | 2.9 | 0.9 | 1.7 | 1.0 | 1.1 | 0.5 | 0.1 | 1.7 | 2.9 | *1.4* |
| Hongkong | 0.0 | 0.0 | 0.0 | 0.0 | 0.1 | 0.1 | 0.3 | 0.2 | 0.1 | 0.4 | 0.4 | 0.4 | 0.2 | 0.0 | 0.2 | 0.1 | *0.3* |
| Macau | 0.0 | 0.0 | 0.0 | 0.1 | 0.0 | 0.3 | 0.2 | 0.2 | 0.2 | 0.5 | 0.2 | 0.2 | 0.2 | 0.0 | 0.5 | 0.2 | *0.3* |
| Taipei | 0.1 | 0.1 | 0.1 | 0.1 | 0.0 | 0.2 | 0.3 | 0.2 | 0.1 | 0.2 | 0.1 | 0.2 | 0.2 | 0.1 | 0.4 | 0.1 | *0.4* |
| Taichun | 0.1 | 0.1 | 0.1 | 0.1 | 0.0 | 0.2 | 0.2 | 0.1 | 0.1 | 0.2 | 0.1 | 0.2 | 0.2 | 0.1 | 0.3 | 0.0 | *0.4* |
| Miaoli | 2.3 | 0.6 | 1.2 | 1.9 | 3.1 | 2.8 | 2.0 | 1.9 | 1.1 | 1.3 | 1.2 | 1.1 | 1.2 | 0.9 | 1.6 | 2.0 | *2.7* |
| Hsinchu | 0.0 | 0.0 | 0.0 | 0.0 | 0.0 | 0.1 | 0.2 | 0.2 | 0.1 | 0.1 | 0.3 | 0.1 | 0.1 | 0.0 | 0.2 | 0.2 | *0.2* |
| Haikou | 0.0 | 0.0 | 0.0 | 0.0 | 0.1 | 0.5 | 0.4 | 1.3 | 0.1 | 0.5 | 0.3 | 0.5 | 0.4 | 0.1 | 0.2 | 0.5 | *0.1* |
| **SC Mean** | **1.2** | **0.9** | **0.5** | **1.8** | **0.9** | **4.3** | **4.8** | **3.9** | **2.5** | **4.1** | **3.0** | **2.8** | **2.4** | **1.1** | **3.5** | **3.1** | ***4.6*** |

**Table S4** Health risk [parameters](file:///D:/Users/Administrator/AppData/Local/Youdao/Dict/8.6.2.0/resultui/html/index.html#/javascript:;) of the four [age](file:///D:/Users/Administrator/AppData/Local/Youdao/Dict/8.6.2.0/resultui/html/index.html#/javascript:;) [group](file:///D:/Users/Administrator/AppData/Local/Youdao/Dict/8.6.2.0/resultui/html/index.html#/javascript:;)s in China

| Exposure parameters | Average lifespan (a) | Inhalation volume (L/min) | | | | Inhalation time (min/d) | | | | Body weight (kg) | | | |
| --- | --- | --- | --- | --- | --- | --- | --- | --- | --- | --- | --- | --- | --- |
|  |  | < 6 | 6-12 | 12-18 | > 18 | < 6 | 6-12 | 12-18 | > 18 | < 6 | 6-12 | 12-18 | > 18 |
| Heilongjiang | 75.98 | 7.4 | 10.5 | 12.0 | 8.4 | 31 | 69 | 76 | 210 | 20.4 | 42.2 | 55.9 | 63.3 |
| Jilin | 76.18 | 7.5 | 10.2 | 12.9 | 8.5 | 155 | 115 | 117 | 146 | 21.1 | 41.2 | 60.5 | 63.5 |
| Liaoning | 76.38 | 7.7 | 10.3 | 11.7 | 8.5 | 81 | 96 | 79 | 185 | 21.9 | 40.7 | 56.8 | 65.0 |
| Beijing | 80.18 | 7.6 | 10 | 11.6 | 8.4 | 83 | 80 | 109 | 195 | 21.8 | 39.2 | 55.0 | 66.9 |
| Hebei | 74.97 | 7.5 | 9.9 | 12.3 | 8.4 | 99 | 94 | 97 | 182 | 21.5 | 38.3 | 57.0 | 65.1 |
| Henan | 74.57 | 7.1 | 9.8 | 12.6 | 8.2 | 162 | 97 | 91 | 300 | 18.8 | 38.7 | 57.9 | 62.8 |
| Inner Mongolia | 74.44 | 7.4 | 9.9 | 12.7 | 8.6 | 82 | 96 | 79 | 250 | 20.3 | 38.6 | 59.9 | 64.8 |
| Shandong | 76.46 | 7.6 | 9.9 | 12.4 | 8.4 | 84 | 79 | 55 | 169 | 21.9 | 38.9 | 59.1 | 65.0 |
| Shanxi | 74.92 | 7.4 | 9.9 | 12.5 | 8.4 | 161 | 103 | 114 | 225 | 20.9 | 39.1 | 57.3 | 64.0 |
| Tianjin | 78.89 | 7.4 | 10.4 | 12.7 | 8.5 | 118 | 75 | 76 | 188 | 20.7 | 41.6 | 60.6 | 65.7 |
| Gansu | 72.23 | 7.0 | 9.7 | 11.7 | 8.2 | 126 | 97 | 117 | 330 | 18.3 | 38.9 | 52.3 | 61.8 |
| Shaanxi | 74.68 | 7.2 | 9.6 | 12.1 | 7.9 | 199 | 119 | 109 | 270 | 19.5 | 36.0 | 55.1 | 59.0 |
| Ningxia | 73.38 | 7.1 | 9.2 | 12.4 | 8.4 | 131 | 100 | 100 | 165 | 18.7 | 32.7 | 57.2 | 62.7 |
| Qinghai | 69.96 | 7.0 | 8.6 | 11.5 | 8.4 | 186 | 127 | 134 | 169 | 18.3 | 28.3 | 50.8 | 62.0 |
| Xinjiang | 72.35 | 7.1 | 9.1 | 12.2 | 8.5 | 204 | 227 | 149 | 285 | 19.0 | 32.3 | 55.1 | 62.4 |
| Yunnan | 69.54 | 6.8 | 9.7 | 11.9 | 8.1 | 183 | 78 | 70 | 320 | 17.5 | 37.2 | 53.4 | 55.9 |
| Chongqing | 75.7 | 7.1 | 9.1 | 11.9 | 7.8 | 162 | 148 | 125 | 207 | 19.2 | 32.2 | 53.3 | 57.3 |
| Sichuan | 74.75 | 7.1 | 9.5 | 12.3 | 8.1 | 93 | 89 | 86 | 249 | 18.9 | 35.9 | 55.0 | 58.2 |
| Tibet^#^ | 68.17 | 6.8 | 9.7 | 11.9 | 8.0 | 167 | 89 | 67 | 339 | 17.5 | 37.2 | 53.4 | 55.1 |
| Guizhou | 71.10 | 7.2 | 9.6 | 11.7 | 8.2 | 95 | 116 | 111 | 229 | 19.3 | 35.3 | 51.5 | 56.0 |
| Anhui | 75.08 | 7.5 | 9.8 | 12.3 | 8.1 | 158 | 140 | 108 | 206 | 21.2 | 36.7 | 55.9 | 60.5 |
| Hubei | 74.87 | 7.2 | 9.2 | 11.9 | 8.3 | 155 | 223 | 103 | 265 | 19.3 | 33.4 | 54.0 | 60.1 |
| Hunan | 74.70 | 7.1 | 9.1 | 12.1 | 8.3 | 202 | 109 | 92 | 210 | 18.8 | 32.5 | 54.7 | 57.3 |
| Jiangsu | 76.63 | 7.4 | 9.9 | 12.3 | 8.1 | 127 | 80 | 68 | 170 | 20.5 | 38.4 | 57.4 | 62.0 |
| Jiangxi | 74.33 | 7.2 | 9.5 | 11.6 | 8 | 124 | 149 | 81 | 227 | 19.7 | 35.0 | 51.6 | 55.9 |
| Shanghai | 80.26 | 7.4 | 10.1 | 12.5 | 8.3 | 160 | 82 | 101 | 155 | 20.6 | 39.5 | 58.1 | 62.2 |
| Zhejiang | 77.73 | 7.1 | 9.3 | 12 | 8.2 | 143 | 85 | 92 | 192 | 19.0 | 34.6 | 54.3 | 59.5 |
| Fujian | 75.76 | 7.1 | 9.4 | 11.9 | 8.1 | 143 | 73 | 79 | 186 | 18.9 | 35.7 | 53.3 | 57.4 |
| Guangxi | 75.11 | 7.0 | 9.2 | 11.2 | 7.9 | 101 | 99 | 88 | 290 | 18.1 | 33.4 | 49.4 | 55.0 |
| Guangdong | 76.49 | 6.8 | 9.2 | 11.7 | 7.9 | 130 | 78 | 84 | 210 | 17.4 | 33.6 | 51.4 | 57.0 |
| Hongkong | 76.49 | 6.8 | 9.2 | 11.7 | 7.9 | 130 | 78 | 84 | 210 | 17.4 | 33.6 | 51.4 | 57.0 |
| Macau | 76.49 | 6.8 | 9.2 | 11.7 | 7.9 | 130 | 78 | 84 | 210 | 17.4 | 33.6 | 51.4 | 57.0 |
| Taiwan | 75.76 | 7.1 | 9.4 | 11.9 | 8.1 | 143 | 73 | 79 | 186 | 18.9 | 35.7 | 53.3 | 57.4 |
| Hainan | 76.30 | 6.9 | 9.1 | 11.6 | 8.0 | 147 | 78 | 96 | 334 | 18.1 | 33.1 | 50.8 | 54.0 |

**Table S5** Ratios between PAH concentration, population, and GDP

|  | NC | CC | SC |
| --- | --- | --- | --- |
| PAH concentration (ng/m^3^) | 241.3 | 33.0 | 54.0 |
| Population (*10^6^ people) | 5.4 | 7.4 | 4.6 |
| GDP (*10^10^ yuan) | 437.2 | 840.3 | 706.2 |
| PAH concentration/population (ng/m^3^/10^6^ people) | 44.7 | 4.5 | 11.7 |
| PAH concentration/GDP (ng/m^3^/10^9^ yuan) | 5.5 | 0.4 | 0.8 |
| GDP/population (10^4^ yuan/people) | 81.0 | 113.6 | 153.5 |

**Table S6** Pearson correlations between PAH content in urban PM and geographical latitude, GDP and population.

|  | Geographical latitude | GDP | Population |
| --- | --- | --- | --- |
| PAHs | 0.435 | -0.162 | -0.068 |

**Table S7** PAH ecological risk.

| PAH | NaP | Acy | Ace | Flu | Ant | Phe | Fla | Pyr | BaA | Chr | BaP | DA | ERM-Q |
| --- | --- | --- | --- | --- | --- | --- | --- | --- | --- | --- | --- | --- | --- |
| Harbin | 0.6 | 1.3 | 0.3 | 0.6 | 0.8 | 2.8 | 0.9 | 1.6 | 1.8 | 0.3 | 0.2 | 1.9 | 1.1 |
| Jiamusi | 0.0 | 0.2 | 0.0 | 0.1 | 0.1 | 0.5 | 0.8 | 1.7 | 2.8 | 1.8 | 2.1 | 2.1 | 1.0 |
| Changchun | 0.0 | 0.0 | 0.6 | 10.3 | 3.6 | 3.8 | 0.0 | 7.2 | 2.4 | 0.4 | 0.8 | 4.3 | 2.8 |
| Jilin | 0.6 | 0.0 | 1.4 | 3.0 | 3.5 | 13.5 | 21.2 | 33.3 | 95.0 | 93.9 | 38.5 | 45.8 | 29.1 |
| Siping | 0.7 | 0.0 | 1.7 | 1.9 | 2.1 | 10.4 | 8.5 | 14.7 | 30.6 | 22.3 | 13.9 | 10.2 | 9.8 |
| Tonghua | 0.5 | 0.0 | 1.3 | 1.9 | 2.9 | 14.1 | 12.4 | 22.2 | 45.3 | 35.7 | 25.9 | 12.8 | 14.6 |
| Baicheng | 0.3 | 0.0 | 0.7 | 1.1 | 1.6 | 7.3 | 8.0 | 12.8 | 23.5 | 17.8 | 13.8 | 2.6 | 7.5 |
| Shenyang | 2.0 | 0.0 | 6.0 | 0.0 | 29.4 | 54.1 | 32.0 | 54.6 | 21.5 | 15.7 | 11.6 | 62.7 | 24.1 |
| Dalian | 0.7 | 0.0 | 1.8 | 0.0 | 0.9 | 4.7 | 2.5 | 3.1 | 3.8 | 2.7 | 3.4 | 25.6 | 4.1 |
| Jinzhou | 0.4 | 0.0 | 2.3 | 0.0 | 2.4 | 5.5 | 7.1 | 8.7 | 9.8 | 3.5 | 6.3 | 46.9 | 7.7 |
| Fushun | 0.5 | 0.0 | 3.2 | 0.0 | 8.7 | 31.7 | 61.8 | 122.3 | 65.0 | 40.4 | 18.5 | 105.4 | 38.1 |
| Anshan | 0.5 | 0.0 | 229.7 | 0.0 | 6.0 | 12.9 | 14.9 | 28.0 | 20.0 | 11.5 | 18.7 | 250.4 | 49.4 |
| Panjing | 0.3 | 0.0 | 1.7 | 0.0 | 1.7 | 3.7 | 4.7 | 5.8 | 6.6 | 2.4 | 4.3 | 31.5 | 5.2 |
| Beijing | 0.1 | 0.1 | 0.8 | 0.9 | 0.5 | 2.4 | 1.2 | 1.9 | 2.6 | 2.0 | 5.1 | 22.0 | 3.3 |
| Shijiazhuang | 0.0 | 0.2 | 0.0 | 0.0 | 1.8 | 0.1 | 0.4 | 0.5 | 1.0 | 1.6 | 4.1 | 8.6 | 1.5 |
| Zhangjiakou | 0.1 | 0.6 | 0.2 | 0.5 | 0.4 | 1.0 | 0.9 | 2.6 | 3.3 | 2.7 | 3.0 | 5.2 | 1.7 |
| Baoding | 1.0 | 7.1 | 4.8 | 8.9 | 1.6 | 3.3 | 0.9 | 1.2 | 4.7 | 1.4 | 2.4 | 12.6 | 4.2 |
| Tangshan | 0.0 | 0.0 | 0.0 | 0.0 | 0.0 | 0.0 | 0.8 | 1.2 | 1.6 | 1.3 | 2.2 | 14.2 | 1.8 |
| Handan | 0.0 | 0.0 | 0.0 | 0.0 | 1.4 | 3.0 | 1.3 | 1.9 | 1.2 | 1.6 | 2.1 | 0.0 | 1.0 |
| Hengshui | 1.9 | 1.7 | 2.8 | 10.5 | 1.6 | 4.9 | 2.5 | 4.7 | 19.4 | 7.5 | 9.7 | 3.7 | 5.9 |
| Zhengzhou | 1.5 | 2.3 | 2.6 | 2.7 | 7.0 | 4.7 | 3.8 | 5.8 | 9.7 | 7.3 | 6.3 | 19.7 | 6.1 |
| Xinxiang | 0.1 | 0.0 | 0.0 | 0.6 | 1.5 | 2.3 | 2.4 | 4.1 | 5.6 | 4.6 | 2.8 | 1.8 | 2.2 |
| Hohhot | 5.8 | 2.1 | 1.5 | 0.4 | 0.3 | 0.4 | 0.0 | 0.8 | 1.2 | 0.8 | 1.2 | 12.9 | 2.3 |
| Baotou | 0.6 | 1.3 | 0.4 | 2.4 | 2.0 | 6.6 | 4.8 | 8.6 | 22.9 | 11.8 | 12.4 | 14.0 | 7.3 |
| Ordos | 0.1 | 0.0 | 0.0 | 0.2 | 0.1 | 0.4 | 0.5 | 0.8 | 1.1 | 1.1 | 1.1 | 2.8 | 0.7 |
| Jinan | 0.5 | 2.6 | 0.2 | 0.3 | 0.5 | 0.8 | 0.8 | 1.2 | 2.0 | 2.4 | 1.9 | 3.0 | 1.4 |
| Qingdao | 0.1 | 0.1 | 0.1 | 0.5 | 0.1 | 1.8 | 1.1 | 1.5 | 1.0 | 1.8 | 1.0 | 0.9 | 0.8 |
| Taiyuan | 0.0 | 0.0 | 0.6 | 0.0 | 1.5 | 6.2 | 3.5 | 8.0 | 4.4 | 2.6 | 4.0 | 2.5 | 2.8 |
| Jinzhong | 0.6 | 1.4 | 2.0 | 8.3 | 10.8 | 48.7 | 37.8 | 46.5 | 36.7 | 52.9 | 34.6 | 36.3 | 26.4 |
| Tianjin | 0.0 | 0.0 | 0.0 | 36.1 | 50.9 | 97.2 | 16.6 | 18.2 | 22.1 | 19.3 | 16.4 | 51.2 | 27.3 |
| Lanzhou | 0.3 | 0.0 | 0.2 | 0.7 | 1.0 | 0.0 | 0.1 | 0.2 | 0.2 | 0.1 | 0.1 | 0.3 | 0.3 |
| Jinchang | 0.0 | 0.0 | 0.0 | 5.6 | 0.0 | 1.2 | 0.6 | 1.4 | 3.4 | 2.9 | 3.0 | 11.9 | 2.5 |
| Xi'an | 0.7 | 6.6 | 3.5 | 3.4 | 2.7 | 2.1 | 1.3 | 1.2 | 6.2 | 1.1 | 2.4 | 14.5 | 3.8 |
| Yulin | 0.0 | 0.0 | 0.0 | 13.6 | 0.0 | 1.4 | 1.4 | 2.8 | 4.7 | 1.8 | 4.0 | 4.0 | 2.8 |
| Yinchuan | 5.7 | 0.3 | 0.7 | 0.0 | 1.1 | 10.4 | 1.7 | 2.4 | 6.2 | 2.0 | 3.4 | 13.1 | 3.9 |
| Xining | 0.0 | 0.0 | 0.0 | 0.0 | 1.9 | 6.0 | 5.8 | 3.7 | 3.2 | 1.4 | 1.0 | 0.0 | 1.9 |
| Urumqi | 12.0 | 60.9 | 30.4 | 60.9 | 7.8 | 27.7 | 13.1 | 27.6 | 33.4 | 28.2 | 4.1 | 198.5 | 42.1 |
| Hotan | 1.3 | 0.6 | 2.7 | 1.2 | 1.3 | 2.1 | 2.6 | 4.5 | 5.1 | 2.6 | 4.0 | 12.1 | 3.3 |
| Changji | 3.0 | 0.3 | 0.2 | 1.3 | 1.6 | 3.4 | 1.8 | 2.7 | 3.3 | 2.1 | 2.6 | 5.4 | 2.3 |
| Karamay | 0.0 | 0.0 | 0.0 | 0.7 | 0.2 | 1.1 | 1.3 | 3.0 | 1.0 | 0.7 | 0.7 | 0.3 | 0.8 |
| Shihezi | 7.6 | 49.4 | 6.4 | 60.6 | 9.6 | 51.5 | 7.0 | 8.5 | 5.6 | 3.6 | 3.6 | 5.9 | 18.3 |
| **ERM-Q ≥ 1.5** | **7** | **8** | **16** | **15** | **24** | **30** | **23** | **31** | **34** | **31** | **33** | **36** | **33** |
| 0.5 ≤ ERM-Q < 1.5 | 13 | 5 | 7 | 9 | 7 | 6 | 13 | 9 | 6 | 7 | 6 | 1 | 7 |
| ERM-Q < 0.5 | 21 | 28 | 18 | 17 | 10 | 5 | 5 | 1 | 1 | 3 | 2 | 4 | 1 |
|  |  |  |  |  |  |  |  |  |  |  |  |  |  |
| Kunming | 0.1 | 1.9 | 0.0 | 0.1 | 0.2 | 1.4 | 0.4 | 1.2 | 1.2 | 1.2 | 1.9 | 0.3 | 0.8 |
| Yuxi | 0.1 | 1.2 | 0.5 | 1.3 | 0.0 | 0.3 | 0.3 | 0.6 | 0.4 | 0.4 | 0.7 | 0.3 | 0.5 |
| Chongqing | 0.3 | 1.0 | 0.0 | 1.2 | 0.7 | 0.6 | 0.5 | 0.7 | 3.7 | 3.7 | 5.2 | 26.3 | 3.7 |
| Chengdu | 0.0 | 0.0 | 0.0 | 0.3 | 0.2 | 0.4 | 0.4 | 0.7 | 1.3 | 0.8 | 1.1 | 0.7 | 0.5 |
| Mianyang | 0.1 | 0.2 | 0.0 | 1.0 | 0.1 | 0.7 | 0.4 | 0.6 | 0.7 | 1.4 | 1.3 | 1.2 | 0.6 |
| Lhasa | 3.7 | 4.7 | 1.0 | 17.6 | 2.0 | 19.4 | 1.5 | 2.3 | 0.8 | 0.7 | 0.7 | 0.9 | 4.6 |
| Guiyang | 0.8 | 10.5 | 9.5 | 1.5 | 1.3 | 3.8 | 0.2 | 0.5 | 1.0 | 0.4 | 0.9 | 7.7 | 3.2 |
| Hefei | 0.0 | 0.1 | 0.0 | 0.0 | 0.5 | 0.4 | 0.2 | 0.5 | 0.3 | 1.1 | 0.9 | 26.1 | 2.5 |
| Huainan | 0.1 | 2.1 | 1.2 | 2.2 | 0.7 | 0.9 | 0.5 | 1.0 | 2.4 | 1.2 | 0.6 | 6.8 | 1.6 |
| Tongling | 0.4 | 2.0 | 1.9 | 2.3 | 2.5 | 1.4 | 0.9 | 1.5 | 3.0 | 1.7 | 2.8 | 15.7 | 3.0 |
| Wuhan | 0.5 | 0.1 | 0.0 | 0.1 | 0.2 | 1.8 | 0.4 | 0.8 | 0.9 | 1.1 | 1.2 | 0.8 | 0.7 |
| Yichang | 0.1 | 0.0 | 0.0 | 0.0 | 0.1 | 0.4 | 0.3 | 0.5 | 1.4 | 0.6 | 1.1 | 0.7 | 0.5 |
| Huangshi | 0.2 | 0.1 | 0.0 | 0.0 | 0.0 | 0.5 | 0.3 | 0.5 | 0.5 | 0.3 | 0.3 | 2.0 | 0.4 |
| Changsha | 0.1 | 4.8 | 0.2 | 2.8 | 1.4 | 0.7 | 0.4 | 0.6 | 0.5 | 0.5 | 1.1 | 3.7 | 1.4 |
| Xiangtan | 0.0 | 0.0 | 0.5 | 2.1 | 0.7 | 1.9 | 0.3 | 0.3 | 0.6 | 0.6 | 1.1 | 1.8 | 0.8 |
| Nanjing | 0.2 | 0.1 | 0.1 | 0.4 | 0.1 | 0.7 | 0.5 | 0.7 | 1.1 | 1.0 | 1.3 | 3.6 | 0.8 |
| Changzhou | 1.2 | 0.1 | 0.2 | 1.1 | 0.2 | 1.0 | 0.4 | 0.8 | 1.5 | 1.3 | 1.7 | 2.1 | 1.0 |
| Xuzhou | 2.4 | 9.9 | 3.8 | 4.0 | 6.8 | 5.0 | 4.8 | 8.8 | 5.0 | 8.3 | 6.8 | 39.6 | 8.8 |
| Wuxi | 0.2 | 0.3 | 0.4 | 0.2 | 0.2 | 0.4 | 0.2 | 0.4 | 1.0 | 0.3 | 0.8 | 2.5 | 0.6 |
| Zhenjiang | 0.1 | 0.5 | 0.4 | 0.1 | 0.0 | 0.4 | 0.2 | 0.5 | 0.4 | 0.3 | 0.6 | 1.7 | 0.4 |
| Nantong | 0.0 | 0.0 | 0.0 | 0.0 | 0.0 | 0.3 | 0.3 | 0.5 | 0.6 | 0.5 | 1.1 | 1.0 | 0.4 |
| Nanchang | 1.0 | 0.0 | 1.3 | 3.5 | 0.2 | 2.0 | 0.2 | 0.3 | 7.5 | 0.5 | 1.8 | 0.0 | 1.5 |
| Jiujiang | 0.0 | 0.0 | 1.0 | 2.8 | 0.3 | 0.8 | 0.2 | 0.1 | 2.8 | 0.1 | 0.9 | 0.0 | 0.8 |
| Shanghai | 0.9 | 2.0 | 0.3 | 1.1 | 0.9 | 2.1 | 0.8 | 1.1 | 2.1 | 0.9 | 1.9 | 1.6 | 1.3 |
| Hangzhou | 0.4 | 1.9 | 1.4 | 2.0 | 1.5 | 0.6 | 0.2 | 0.5 | 1.1 | 0.7 | 1.1 | 9.2 | 1.7 |
| Huzhou | 0.0 | 0.0 | 0.1 | 0.0 | 0.1 | 0.4 | 0.3 | 0.6 | 0.3 | 0.5 | 0.4 | 0.5 | 0.3 |
| Ningbo | 0.0 | 0.0 | 0.0 | 0.0 | 0.2 | 0.3 | 0.2 | 0.4 | 0.5 | 0.5 | 0.6 | 2.5 | 0.4 |
| Wenzhou | 0.2 | 2.5 | 1.3 | 1.9 | 0.5 | 2.7 | 0.6 | 0.8 | 2.3 | 0.8 | 1.2 | 1.0 | 1.3 |
| Taizhou | 0.1 | 0.1 | 0.2 | 0.3 | 0.1 | 0.6 | 0.2 | 0.4 | 0.7 | 0.7 | 1.3 | 3.7 | 0.7 |
| Jinhua | 0.0 | 0.2 | 0.2 | 0.2 | 0.1 | 0.1 | 0.0 | 0.1 | 0.3 | 0.3 | 0.5 | 1.2 | 0.3 |
| ERM-Q ≥ 1.5 | 2 | 10 | 3 | 11 | 3 | 8 | 2 | 3 | 9 | 3 | 7 | 17 | 9 |
| **0.5 ≤ ERM-Q < 1.5** | **4** | **3** | **7** | **5** | **9** | **12** | **4** | **16** | **14** | **16** | **21** | **8** | **13** |
| ERM-Q < 0.5 | 24 | 17 | 20 | 14 | 18 | 10 | 24 | 11 | 7 | 11 | 2 | 5 | 8 |
|  |  |  |  |  |  |  |  |  |  |  |  |  |  |
| Xiamen | 0.0 | 0.0 | 0.0 | 0.0 | 0.1 | 0.0 | 0.0 | 0.1 | 0.4 | 0.2 | 0.3 | 1.5 | 0.2 |
| Longyan | 1.2 | 23.6 | 0.0 | 8.5 | 1.3 | 60.7 | 16.1 | 26.5 | 1.1 | 24.0 | 4.3 | 0.0 | 13.9 |
| Nanning | 6.9 | 0.9 | 1.5 | 15.0 | 0.6 | 11.9 | 1.1 | 3.6 | 0.7 | 0.9 | 0.1 | 0.0 | 3.6 |
| Baize | 0.9 | 0.0 | 0.6 | 1.1 | 0.4 | 1.1 | 0.4 | 0.8 | 1.3 | 1.3 | 1.2 | 5.6 | 1.2 |
| Liuzhou | 1.4 | 4.1 | 3.1 | 3.5 | 5.0 | 3.3 | 2.1 | 3.2 | 11.5 | 4.7 | 6.7 | 5.8 | 4.5 |
| Guangzhou | 0.0 | 0.1 | 0.0 | 0.3 | 0.1 | 0.3 | 0.1 | 0.3 | 0.3 | 0.3 | 0.5 | 0.6 | 0.2 |
| Shenzhen | 0.3 | 0.1 | 0.0 | 0.2 | 0.1 | 0.4 | 0.2 | 0.4 | 0.4 | 0.6 | 0.8 | 1.6 | 0.4 |
| Qingyuan | 0.0 | 0.1 | 0.0 | 0.1 | 0.1 | 0.6 | 0.5 | 0.8 | 0.6 | 0.7 | 2.1 | 8.6 | 1.2 |
| Shaoguan | 1.2 | 3.5 | 2.3 | 3.5 | 4.3 | 3.2 | 1.9 | 3.0 | 10.2 | 0.0 | 6.3 | 6.2 | 3.8 |
| Shantou | 1.6 | 0.0 | 0.1 | 0.0 | 0.4 | 1.7 | 0.5 | 2.6 | 1.8 | 3.1 | 5.5 | 5.8 | 1.9 |
| Dongguan | 0.0 | 6.5 | 1.7 | 75.9 | 0.1 | 0.0 | 5.1 | 1.1 | 0.6 | 0.6 | 0.3 | 0.5 | 7.7 |
| Hongkong | 0.0 | 0.1 | 0.0 | 0.0 | 0.1 | 0.1 | 0.1 | 0.1 | 0.1 | 0.1 | 0.1 | 0.1 | 0.1 |
| Macau | 0.0 | 0.0 | 0.0 | 0.2 | 0.0 | 0.2 | 0.0 | 0.1 | 0.1 | 0.2 | 0.1 | 0.0 | 0.1 |
| Taipei | 0.1 | 0.2 | 0.1 | 0.2 | 0.0 | 0.1 | 0.1 | 0.1 | 0.1 | 0.1 | 0.2 | 0.3 | 0.1 |
| Taichun | 0.1 | 0.2 | 0.1 | 0.2 | 0.0 | 0.1 | 0.0 | 0.1 | 0.1 | 0.1 | 0.1 | 0.3 | 0.1 |
| Miaoli | 1.1 | 1.2 | 1.9 | 3.6 | 2.8 | 1.9 | 0.4 | 0.7 | 0.7 | 0.5 | 0.8 | 3.3 | 1.6 |
| Hsinchu | 0.0 | 0.0 | 0.0 | 0.0 | 0.0 | 0.1 | 0.0 | 0.1 | 0.0 | 0.0 | 0.1 | 0.1 | 0.0 |
| Haikou | 0.0 | 0.0 | 0.0 | 0.0 | 0.1 | 0.3 | 0.1 | 0.5 | 0.1 | 0.2 | 0.2 | 0.2 | 0.1 |
| ERM-Q ≥ 1.5 | 2 | 4 | 4 | 6 | 3 | 6 | 4 | 5 | 3 | 3 | 5 | 8 | 7 |
| 0.5 ≤ ERM-Q < 1.5 | 5 | 2 | 2 | 1 | 2 | 2 | 2 | 5 | 6 | 5 | 3 | 1 | 2 |
| **ERM-Q < 0.5** | **11** | **12** | **12** | **11** | **13** | **10** | **12** | **8** | **9** | **10** | **10** | **9** | **9** |

**Table S8** Concentrations (ng/m^3^) and proportions (%) of PAHs.

| Province | City | LMW PAHs | LMW PAHs (%) | MMW PAHs | MMW PAHs (%) | HMW PAHs | HMW PAHs (%) | CAN-PAHs | CAN-PAHs (%) | NCAN-PAHs | NCAN-PAHs (%) | COM-PAHs | COM-PAHs (%) | LMW PAHs/HMW PAHs | （LMW+MMW PAHs）/HMW PAHs |
| --- | --- | --- | --- | --- | --- | --- | --- | --- | --- | --- | --- | --- | --- | --- | --- |
| Heilongjiang | Harbin | 7.6 | 32.0 | 12.3 | 52.2 | 3.7 | 15.8 | 7.1 | 30.0 | 16.5 | 70.0 | 15.6 | 65.9 | 2.0 | **5.4** |
|  | Jiamusi | 1.1 | 3.4 | 18.0 | 54.9 | 13.7 | 41.7 | 21.4 | 65.2 | 11.4 | 34.8 | 31.2 | 94.9 | 0.1 | **1.4** |
| Jilin | Changchun | 15.6 | 28.2 | 23.6 | 42.7 | 16.1 | 29.1 | 17.1 | 30.9 | 38.1 | 69.1 | 38.5 | 69.7 | 1.0 | **2.4** |
|  | Jilin | 27.7 | 2.3 | 609.6 | 50.7 | 564.1 | 47.0 | 937.1 | 78.0 | 264.3 | 22.0 | 1161.8 | 96.7 | 0.0 | **1.1** |
|  | Siping | 21.7 | 5.7 | 192.8 | 50.5 | 167.7 | 43.9 | 258.3 | 67.6 | 123.9 | 32.4 | 357.8 | 93.6 | 0.1 | **1.3** |
|  | Tonghua | 27.2 | 4.5 | 293.1 | 48.9 | 278.8 | 46.5 | 414.0 | 69.1 | 185.1 | 30.9 | 568.6 | 94.9 | 0.1 | **1.1** |
|  | Baicheng | 14.3 | 4.2 | 161.5 | 47.2 | 166.7 | 48.7 | 236.5 | 69.0 | 106.0 | 31.0 | 327.5 | 95.6 | 0.1 | **1.1** |
| Liaoning | Shenyang | 121.4 | 20.1 | 383.3 | 63.5 | 99.3 | 16.4 | 174.5 | 28.9 | 429.4 | 71.1 | 466.3 | 77.2 | 1.2 | **5.1** |
|  | Dalian | 10.7 | 14.2 | 34.3 | 45.5 | 30.3 | 40.2 | 42.4 | 56.2 | 33.0 | 43.8 | 57.9 | 76.9 | 0.4 | **1.5** |
|  | Jinzhou | 13.1 | 8.7 | 84.1 | 56.0 | 53.0 | 35.3 | 76.2 | 50.8 | 73.9 | 49.2 | 124.9 | 83.2 | 0.2 | **1.8** |
|  | Fushun | 60.4 | 5.6 | 850.0 | 78.6 | 170.7 | 15.8 | 381.2 | 35.3 | 699.9 | 64.7 | 993.3 | 91.9 | 0.4 | **5.3** |
|  | Anshan | 174.0 | 28.9 | 213.1 | 35.4 | 215.5 | 35.8 | 242.7 | 40.3 | 359.9 | 59.7 | 363.5 | 60.3 | 0.8 | **1.8** |
|  | Panjing | 9.1 | 9.0 | 56.3 | 55.5 | 36.0 | 35.5 | 51.4 | 50.7 | 50.0 | 49.3 | 84.1 | 82.9 | 0.3 | **1.8** |
| Beijing | Beijing | 5.6 | 8.5 | 20.9 | 31.5 | 39.8 | 60.0 | 43.3 | 65.4 | 22.9 | 34.6 | 54.9 | 82.9 | 0.1 | **0.7** |
| Hebei | Shijiazhuang | 2.2 | 6.7 | 9.5 | 28.9 | 21.1 | 64.4 | 24.9 | 75.9 | 7.9 | 24.1 | 28.3 | 86.4 | 0.1 | **0.6** |
|  | Zhangjiakou | 3.0 | 6.1 | 24.0 | 49.1 | 21.9 | 44.8 | 31.7 | 64.9 | 17.1 | 35.1 | 44.5 | 91.1 | 0.1 | **1.2** |
|  | Baoding | 20.2 | 30.7 | 18.8 | 28.6 | 26.8 | 40.7 | 34.2 | 52.0 | 31.6 | 48.0 | 42.3 | 64.3 | 0.8 | **1.5** |
|  | Tangshan | 0.1 | 0.3 | 13.0 | 36.4 | 22.7 | 63.4 | 22.8 | 63.5 | 13.1 | 36.5 | 32.1 | 89.4 | 0.0 | **0.6** |
|  | Handan | 6.0 | 14.6 | 17.8 | 43.6 | 17.1 | 41.8 | 20.8 | 50.8 | 20.1 | 49.2 | 34.9 | 85.4 | 0.3 | **1.4** |
|  | Hengshui | 21.5 | 12.7 | 76.9 | 45.6 | 70.4 | 41.7 | 112.2 | 66.5 | 56.6 | 33.5 | 146.3 | 86.7 | 0.3 | **1.4** |
| Henan | Zhengzhou | 22.2 | 12.5 | 70.1 | 39.5 | 85.2 | 48.0 | 110.0 | 62.0 | 67.4 | 38.0 | 150.1 | 84.6 | 0.3 | **1.1** |
|  | Xinxiang | 5.6 | 6.8 | 44.7 | 54.6 | 31.6 | 38.5 | 49.0 | 59.8 | 32.9 | 40.2 | 75.8 | 92.6 | 0.2 | **1.6** |
| Inner Mongolia | Hohhot | 15.3 | 47.4 | 6.4 | 19.9 | 10.6 | 32.8 | 14.2 | 44.1 | 18.0 | 55.9 | 13.6 | 42.2 | 1.4 | **2.0** |
|  | Baotou | 15.7 | 6.8 | 116.4 | 50.5 | 98.3 | 42.7 | 152.5 | 66.2 | 77.7 | 33.8 | 211.0 | 91.6 | 0.2 | **1.3** |
|  | Dongsheng | 1.1 | 4.1 | 9.3 | 35.4 | 16.0 | 60.6 | 14.8 | 56.0 | 11.6 | 44.0 | 24.6 | 93.2 | 0.1 | **0.7** |
|  | Hangjin | 0.1 | 2.4 | 2.3 | 61.0 | 1.4 | 36.6 | 2.8 | 73.5 | 1.0 | 26.5 | 3.5 | 94.1 | 0.1 | **1.7** |
|  | Jungar Banner | 3.7 | 2.9 | 36.0 | 28.4 | 86.8 | 68.6 | 66.5 | 52.6 | 60.0 | 47.4 | 116.7 | 92.3 | 0.0 | **0.5** |
| Shandong | Jinan | 4.4 | 9.8 | 17.1 | 37.8 | 23.7 | 52.4 | 28.9 | 63.9 | 16.3 | 36.1 | 40.0 | 88.4 | 0.2 | **0.9** |
|  | Qingdao | 3.5 | 11.5 | 16.4 | 54.5 | 10.2 | 34.0 | 15.6 | 51.7 | 14.5 | 48.3 | 26.4 | 87.7 | 0.3 | **2.0** |
| Shanxi | Taiyuan | 11.4 | 13.0 | 52.9 | 60.2 | 23.6 | 26.9 | 30.7 | 34.9 | 57.2 | 65.1 | 75.9 | 86.3 | 0.5 | **2.7** |
|  | Jinzhong | 92.8 | 10.2 | 520.7 | 57.2 | 297.0 | 32.6 | 467.4 | 51.3 | 443.1 | 48.7 | 808.3 | 88.8 | 0.3 | **2.1** |
| Tianjin | Tianjin | 221.3 | 25.6 | 221.5 | 25.6 | 423.0 | 48.9 | 505.0 | 58.3 | 360.8 | 41.7 | 631.2 | 72.9 | 0.5 | **1.0** |
| Gansu | Lanzhou | 2.4 | 44.8 | 1.6 | 30.5 | 1.3 | 24.7 | 1.9 | 34.8 | 3.5 | 65.2 | 2.9 | 53.9 | 1.8 | **3.1** |
|  | Jinchang | 4.8 | 5.6 | 20.2 | 23.6 | 60.7 | 70.8 | 65.8 | 76.8 | 19.9 | 23.2 | 77.8 | 90.8 | 0.1 | **0.4** |
| Shaanxi | Xi'an | 14.9 | 23.6 | 22.8 | 36.1 | 25.4 | 40.3 | 34.6 | 54.8 | 28.6 | 45.2 | 44.4 | 70.4 | 0.6 | **1.5** |
|  | Yulin | 9.4 | 10.0 | 27.3 | 28.9 | 57.6 | 61.1 | 63.1 | 66.9 | 31.2 | 33.1 | 83.8 | 88.9 | 0.2 | **0.6** |
| Ningxia | Yinchuan | 29.3 | 29.9 | 30.2 | 30.8 | 38.5 | 39.3 | 48.0 | 49.0 | 50.0 | 51.0 | 65.3 | 66.6 | 0.8 | **1.5** |
| Qinghai | Xining | 11.1 | 16.2 | 48.0 | 69.9 | 9.6 | 13.9 | 18.0 | 26.1 | 50.8 | 73.9 | 57.6 | 83.8 | 1.2 | **6.2** |
| Xinjiang | Urumqi | 158.1 | 19.7 | 271.2 | 33.8 | 373.2 | 46.5 | 427.6 | 53.3 | 374.9 | 46.7 | 592.8 | 73.9 | 0.4 | **1.2** |
|  | Hotan | 10.0 | 10.7 | 40.5 | 43.3 | 43.0 | 46.0 | 52.4 | 56.0 | 41.1 | 44.0 | 80.3 | 86.0 | 0.2 | **1.2** |
|  | Miquan | 77.8 | 16.5 | 223.4 | 47.5 | 168.8 | 35.9 | 213.8 | 45.5 | 256.1 | 54.5 | 387.1 | 82.4 | 0.5 | **1.8** |
|  | Changji | 14.1 | 19.7 | 27.3 | 38.0 | 30.4 | 42.3 | 36.1 | 50.3 | 35.7 | 49.7 | 56.3 | 78.4 | 0.5 | **1.4** |
|  | Karamay | 2.2 | 7.1 | 17.8 | 57.1 | 11.2 | 35.8 | 12.9 | 41.3 | 18.3 | 58.7 | 28.9 | 92.6 | 0.2 | **1.8** |
|  | Shihezi | 165.3 | 59.9 | 76.7 | 27.8 | 33.8 | 12.3 | 47.6 | 17.3 | 228.3 | 82.7 | 109.0 | 39.5 | 4.9 | **7.2** |
| Yunnan | Kunming | 3.5 | 12.3 | 10.5 | 37.1 | 14.3 | 50.5 | 16.5 | 58.4 | 11.8 | 41.6 | 24.7 | 87.4 | 0.2 | **1.0** |
|  | Yuxi | 2.3 | 17.2 | 4.8 | 35.5 | 6.4 | 47.3 | 6.4 | 47.9 | 7.0 | 52.1 | 11.0 | 82.1 | 0.4 | **1.1** |
| Chongqing | Chongqing | 3.4 | 3.2 | 20.7 | 19.3 | 83.0 | 77.5 | 87.1 | 81.3 | 20.0 | 18.7 | 96.8 | 90.4 | 0.0 | **0.3** |
| Sichuan | Chengdu | 1.0 | 5.1 | 8.0 | 42.5 | 9.8 | 52.4 | 12.7 | 67.9 | 6.0 | 32.1 | 17.6 | 93.9 | 0.1 | **0.9** |
|  | Mianyang | 2.0 | 9.0 | 8.8 | 40.1 | 11.2 | 50.9 | 14.3 | 65.0 | 7.7 | 35.0 | 19.7 | 89.6 | 0.2 | **1.0** |
| Xizang | Lhasa | 51.7 | 68.5 | 16.9 | 22.4 | 6.8 | 9.0 | 8.7 | 11.5 | 66.7 | 88.5 | 23.5 | 31.2 | 7.6 | **10.1** |
|  | Linzhi | 0.1 | 26.7 | 0.1 | 11.1 | 0.3 | 62.2 | 0.3 | 55.6 | 0.2 | 44.4 | 0.3 | 71.1 | 0.4 | **0.7** |
| Guizhou | Guiyang | 21.1 | 55.4 | 4.9 | 12.8 | 12.1 | 31.8 | 11.8 | 31.0 | 26.3 | 69.0 | 15.0 | 39.3 | 1.7 | **2.1** |
| Anhui | Hefei | 1.3 | 3.1 | 5.8 | 14.2 | 33.6 | 82.7 | 20.7 | 51.0 | 19.9 | 49.0 | 32.6 | 80.2 | 0.0 | **0.2** |
|  | Huainan | 5.3 | 17.2 | 12.0 | 38.7 | 13.7 | 44.1 | 17.7 | 57.0 | 13.4 | 43.0 | 24.0 | 77.1 | 0.4 | **1.3** |
|  | Tongling | 9.1 | 16.6 | 18.2 | 33.2 | 27.6 | 50.3 | 32.2 | 58.8 | 22.6 | 41.2 | 41.6 | 76.0 | 0.3 | **1.0** |
| Hubei | Wuhan | 4.1 | 16.1 | 8.9 | 34.6 | 12.7 | 49.3 | 15.2 | 59.1 | 10.5 | 40.9 | 21.3 | 83.1 | 0.3 | **1.0** |
|  | Yichang | 1.1 | 5.8 | 6.8 | 34.8 | 11.7 | 59.4 | 13.4 | 67.9 | 6.3 | 32.1 | 18.3 | 93.3 | 0.1 | **0.7** |
|  | Huangshi | 1.3 | 12.5 | 4.6 | 43.2 | 4.7 | 44.3 | 5.3 | 49.4 | 5.4 | 50.6 | 8.8 | 82.6 | 0.3 | **1.3** |
| Hunan | Changsha | 6.8 | 31.2 | 5.7 | 26.4 | 9.2 | 42.4 | 9.3 | 42.8 | 12.4 | 57.2 | 14.0 | 64.4 | 0.7 | **1.4** |
|  | Xiangtan | 5.1 | 26.8 | 5.1 | 27.0 | 8.8 | 46.2 | 9.9 | 52.2 | 9.1 | 47.8 | 13.4 | 70.7 | 0.6 | **1.2** |
| Jiangsu | Nanjing | 1.9 | 6.9 | 8.8 | 31.5 | 17.3 | 61.6 | 18.3 | 65.2 | 9.8 | 34.8 | 25.2 | 89.7 | 0.1 | **0.6** |
|  | Changzhou | 5.0 | 15.7 | 10.3 | 32.2 | 16.6 | 52.1 | 18.6 | 58.4 | 13.2 | 41.6 | 26.3 | 82.6 | 0.3 | **0.9** |
|  | Xuzhou | 29.6 | 18.0 | 78.8 | 47.9 | 56.2 | 34.1 | 75.3 | 45.8 | 89.3 | 54.2 | 124.7 | 75.8 | 0.5 | **1.9** |
|  | Wuxi | 1.7 | 10.5 | 4.4 | 26.5 | 10.4 | 63.0 | 9.7 | 58.5 | 6.9 | 41.5 | 14.1 | 85.6 | 0.2 | **0.6** |
|  | Zhenjiang | 1.5 | 12.2 | 3.9 | 31.4 | 6.9 | 56.4 | 7.2 | 58.5 | 5.1 | 41.5 | 10.3 | 84.1 | 0.2 | **0.8** |
|  | Nantong | 0.5 | 4.1 | 5.2 | 40.4 | 7.2 | 55.5 | 7.4 | 57.0 | 5.6 | 43.0 | 12.2 | 93.9 | 0.1 | **0.8** |
| Jiangxi | Nanchang | 8.0 | 22.6 | 15.0 | 42.2 | 12.5 | 35.2 | 22.9 | 64.4 | 12.7 | 35.6 | 27.5 | 77.4 | 0.6 | **1.8** |
|  | Jiujiang | 3.8 | 20.7 | 5.8 | 31.3 | 8.9 | 48.0 | 10.5 | 56.9 | 8.0 | 43.1 | 14.6 | 79.3 | 0.4 | **1.1** |
| Shanghai | Shanghai | 7.7 | 23.3 | 12.4 | 37.5 | 13.0 | 39.2 | 16.2 | 48.9 | 16.9 | 51.1 | 25.0 | 75.4 | 0.6 | **1.5** |
| Zhejiang | Hangzhou | 6.2 | 24.5 | 6.3 | 25.0 | 12.7 | 50.5 | 14.1 | 56.1 | 11.0 | 43.9 | 16.6 | 66.0 | 0.5 | **1.0** |
|  | Huzhou | 0.7 | 6.4 | 4.7 | 40.4 | 6.2 | 53.1 | 5.5 | 47.2 | 6.1 | 52.8 | 10.7 | 92.5 | 0.1 | **0.9** |
|  | Ningbo | 0.6 | 5.0 | 4.1 | 31.6 | 8.2 | 63.5 | 8.7 | 67.3 | 4.2 | 32.7 | 11.6 | 90.1 | 0.1 | **0.6** |
|  | Wenzhou | 8.2 | 25.5 | 11.2 | 34.9 | 12.7 | 39.6 | 16.5 | 51.3 | 15.7 | 48.7 | 23.7 | 73.7 | 0.6 | **1.5** |
|  | Taizhou | 1.5 | 7.2 | 5.0 | 24.4 | 14.2 | 68.5 | 13.9 | 66.9 | 6.8 | 33.1 | 18.2 | 88.2 | 0.1 | **0.5** |
|  | Jinhua | 0.7 | 7.7 | 1.8 | 20.9 | 6.1 | 71.4 | 6.7 | 78.4 | 1.9 | 21.6 | 7.6 | 88.6 | 0.1 | **0.4** |
| Fujian | Xiamen | 0.1 | 2.3 | 1.6 | 35.4 | 2.7 | 62.3 | 3.3 | 74.4 | 1.1 | 25.6 | 3.9 | 88.8 | 0.0 | **0.6** |
|  | Longyan | 111.5 | 31.0 | 219.8 | 61.2 | 27.9 | 7.8 | 96.8 | 27.0 | 262.3 | 73.0 | 247.7 | 69.0 | 4.0 | **11.9** |
| Guangxi | Nanning | 42.6 | 66.3 | 18.7 | 29.2 | 2.9 | 4.5 | 6.1 | 9.4 | 58.1 | 90.6 | 21.6 | 33.7 | 14.7 | **21.1** |
|  | Baize | 5.0 | 15.2 | 10.0 | 30.3 | 18.0 | 54.5 | 20.1 | 60.8 | 13.0 | 39.2 | 26.6 | 80.4 | 0.3 | **0.8** |
|  | Liuzhou | 19.1 | 18.6 | 50.5 | 49.2 | 33.2 | 32.3 | 61.9 | 60.2 | 40.9 | 39.8 | 82.2 | 80.0 | 0.6 | **2.1** |
| Guangdong | Guangzhou | 0.7 | 7.5 | 2.7 | 27.6 | 6.3 | 64.8 | 6.4 | 66.1 | 3.3 | 33.9 | 8.8 | 90.9 | 0.1 | **0.5** |
|  | Shenzhen | 1.6 | 8.5 | 4.3 | 23.3 | 12.7 | 68.3 | 10.7 | 57.5 | 7.9 | 42.5 | 16.6 | 89.3 | 0.1 | **0.5** |
|  | Qingyuan | 1.1 | 2.2 | 7.2 | 15.0 | 39.7 | 82.8 | 27.3 | 56.9 | 20.6 | 43.1 | 44.7 | 93.2 | 0.0 | **0.2** |
|  | Shaoguan | 17.2 | 16.8 | 33.7 | 32.9 | 51.6 | 50.3 | 55.6 | 54.3 | 46.8 | 45.7 | 83.6 | 81.6 | 0.3 | **1.0** |
|  | Shantou | 6.3 | 6.2 | 21.0 | 20.4 | 75.3 | 73.4 | 67.2 | 65.5 | 35.4 | 34.5 | 94.7 | 92.4 | 0.1 | **0.4** |
|  | Dongguan | 45.5 | 54.0 | 31.4 | 37.3 | 7.3 | 8.7 | 8.3 | 9.8 | 76.0 | 90.2 | 38.6 | 45.8 | 6.2 | **10.5** |
| Hongkong | Hongkong | 0.2 | 9.5 | 0.9 | 37.9 | 1.3 | 52.6 | 1.6 | 65.5 | 0.8 | 34.5 | 2.2 | 89.0 | 0.2 | **0.8** |
| Macao | Macao | 0.4 | 14.4 | 1.1 | 42.2 | 1.2 | 43.3 | 1.4 | 50.9 | 1.3 | 49.1 | 2.3 | 85.6 | 0.3 | **1.3** |
| Taiwan | Taipei | 0.7 | 25.3 | 0.9 | 33.5 | 1.1 | 41.2 | 1.0 | 39.7 | 1.6 | 60.3 | 1.9 | 72.2 | 0.6 | **1.5** |
|  | Taichun | 0.6 | 25.6 | 0.7 | 31.7 | 1.0 | 42.7 | 1.0 | 43.1 | 1.3 | 56.9 | 1.6 | 71.5 | 0.6 | **1.3** |
|  | Miaoli | 12.0 | 46.0 | 6.2 | 23.8 | 7.9 | 30.2 | 8.7 | 33.3 | 17.5 | 66.7 | 13.3 | 50.7 | 1.5 | **2.3** |
|  | Hsinchu | 0.2 | 10.2 | 0.6 | 36.6 | 0.8 | 53.2 | 0.8 | 53.8 | 0.7 | 46.2 | 1.4 | 88.1 | 0.2 | **1.0** |
| Hainan | Haikou | 0.5 | 11.4 | 2.3 | 48.7 | 1.9 | 39.8 | 2.3 | 48.5 | 2.4 | 51.5 | 4.1 | 87.5 | 0.3 | **1.5** |
| All cities | Mean | 20.7 | 16.9 | 62.0 | 37.9 | 51.2 | 45.2 | 70.3 | 53.5 | 63.5 | 46.5 | 109.5 | 80.0 | 0.8 | **2.0** |
|  | Min | 0.1 | 0.3 | 0.1 | 11.1 | 0.3 | 4.5 | 0.3 | 9.4 | 0.2 | 18.7 | 0.3 | 31.2 | 0.0 | **0.2** |
|  | Max | 221.3 | 68.5 | 850.0 | 78.6 | 564.1 | 82.8 | 937.1 | 81.3 | 699.9 | 90.6 | 1161.8 | 96.7 | 14.7 | **21.1** |
|  | Median | 5.6 | 12.5 | 16.4 | 36.1 | 16.1 | 44.8 | 18.6 | 56.0 | 18.0 | 44.0 | 28.9 | 84.1 | 0.3 | **1.2** |

**Table S9** HIs of PAHs in Chinese urban PM.

| Province | City | < 6 | 6-12 | 12-18 | > 18 | Province | City | < 6 | 6-12 | 12-18 | > 18 |
| --- | --- | --- | --- | --- | --- | --- | --- | --- | --- | --- | --- |
| Heilongjiang | Harbin | 0.1 | 0.5 | 0.6 | 1.1 | Yunnan | Kunming | 2.5 | 1.5 | 1.7 | 5.2 |
|  | Jiamusi | 0.6 | 1.8 | 2.3 | 4.4 |  | Yuxi | 1.0 | 0.6 | 0.6 | 2.0 |
| Jilin | Changchun | 2.2 | 2.2 | 2.8 | 2.3 | Chongqing | Chongqing | 11.9 | 13.9 | 15.4 | 16.7 |
|  | Jilin | 76.3 | 77.0 | 99.0 | 81.4 | Sichuan | Chengdu | 0.9 | 1.2 | 1.5 | 2.8 |
|  | Siping | 24.0 | 24.2 | 31.1 | 25.6 |  | Mianyang | 1.0 | 1.3 | 1.6 | 3.1 |
|  | Tonghua | 40.8 | 41.2 | 53.0 | 43.6 | Tibet | Lhasa | 1.1 | 0.8 | 0.8 | 2.6 |
|  | Baicheng | 22.3 | 22.5 | 28.9 | 23.8 | Guizhou | Guiyang | 1.4 | 2.2 | 2.6 | 3.8 |
| Liaoning | Shenyang | 13.7 | 21.6 | 20.2 | 34.4 | Anhui | Hefei | 5.3 | 6.2 | 6.0 | 7.5 |
|  | Dalian | 4.3 | 6.8 | 6.4 | 10.9 |  | Huainan | 2.3 | 2.6 | 2.6 | 3.2 |
|  | Jinzhou | 8.1 | 12.8 | 11.9 | 20.3 |  | Tongling | 6.0 | 7.0 | 6.8 | 8.5 |
|  | Fushun | 24.0 | 38.0 | 35.5 | 60.4 | Hubei | Wuhan | 1.7 | 3.1 | 1.9 | 3.4 |
|  | Anshan | 32.2 | 51.0 | 47.7 | 81.1 |  | Yichang | 1.6 | 2.9 | 1.7 | 3.1 |
|  | Panjin | 5.4 | 8.6 | 8.1 | 13.7 |  | Huangshi | 0.7 | 1.3 | 0.8 | 1.4 |
| Beijing | Beijing | 5.0 | 6.3 | 10.0 | 12.9 | Hunan | Changsha | 2.2 | 1.5 | 1.7 | 2.7 |
| Hebei | Shijiazhuang | 3.6 | 4.5 | 5.8 | 7.4 |  | Xiangtan | 1.9 | 1.3 | 1.5 | 2.4 |
|  | Zhangjiakou | 2.9 | 3.6 | 4.6 | 5.9 | Jiangsu | Nanjing | 2.0 | 1.7 | 1.7 | 2.9 |
|  | Baoding | 3.4 | 4.3 | 5.5 | 7.0 |  | Changzhou | 2.0 | 1.7 | 1.8 | 2.9 |
|  | Tangshan | 3.1 | 3.8 | 4.9 | 6.3 |  | Xuzhou | 11.1 | 9.4 | 9.9 | 16.3 |
|  | Handan | 1.7 | 2.1 | 2.7 | 3.5 |  | Wuxi | 1.2 | 1.0 | 1.1 | 1.8 |
|  | Hengshui | 8.6 | 10.8 | 13.9 | 17.8 |  | Zhenjiang | 0.8 | 0.7 | 0.8 | 1.2 |
| Henan | Zhengzhou | 12.7 | 10.5 | 12.7 | 27.2 |  | Nantong | 1.1 | 0.9 | 1.0 | 1.6 |
|  | Xinxiang | 4.6 | 3.8 | 4.6 | 9.8 | Jiangxi | Nanchang | 2.0 | 3.2 | 2.1 | 4.1 |
| Inner Mongolia | Hohhot | 1.7 | 2.7 | 2.9 | 6.2 |  | Jiujiang | 1.0 | 1.6 | 1.1 | 2.1 |
|  | Baotou | 9.8 | 15.3 | 16.2 | 34.6 | Shanghai | Shanghai | 2.6 | 1.8 | 2.7 | 2.8 |
|  | Ordos | 1.0 | 1.6 | 1.7 | 3.6 | Zhejiang | Hangzhou | 2.5 | 1.9 | 2.7 | 3.8 |
| Shandong | Jinan | 1.8 | 2.2 | 1.9 | 4.0 |  | Huzhou | 0.5 | 0.4 | 0.6 | 0.8 |
|  | Qingdao | 0.9 | 1.1 | 0.9 | 1.9 |  | Ningbo | 1.1 | 0.8 | 1.2 | 1.7 |
| Shanxi | Taiyuan | 5.1 | 4.4 | 6.1 | 8.1 |  | Wenzhou | 1.7 | 1.3 | 1.8 | 2.6 |
|  | Jinzhong | 52.9 | 45.3 | 63.3 | 84.0 |  | Taizhou | 1.9 | 1.5 | 2.1 | 3.0 |
| Tianjin | Tianjin | 34.4 | 30.7 | 38.0 | 62.9 |  | Jinhua | 0.8 | 0.6 | 0.9 | 1.2 |
| Gansu | Lanzhou | 0.2 | 0.2 | 0.3 | 0.5 | **CC** | **Mean** | **2.5** | **2.5** | **2.6** | **3.9** |
|  | Jinchang | 5.5 | 5.9 | 8.5 | 16.9 | Fujian | Xiamen | 0.5 | 0.3 | 0.5 | 0.7 |
| Shaanxi | Xi'an | 6.9 | 5.5 | 6.4 | 10.3 |  | Longyan | 5.0 | 3.3 | 4.6 | 7.3 |
|  | Yulin | 8.7 | 7.0 | 8.0 | 13.0 | Guangxi | Nanning | 0.2 | 0.3 | 0.3 | 0.7 |
| Ningxia | Yinchuan | 5.5 | 5.5 | 7.4 | 8.3 |  | Baize | 1.6 | 2.1 | 2.3 | 5.3 |
| Qinghai | Xining | 1.8 | 1.5 | 2.2 | 2.0 |  | Liuzhou | 5.5 | 7.0 | 7.6 | 17.7 |
| Xinjiang | Urumqi | 61.9 | 88.2 | 77.7 | 103.5 | Guangdong | Guangzhou | 0.6 | 0.5 | 0.7 | 1.1 |
|  | Hotan | 9.2 | 13.2 | 11.6 | 15.5 |  | Shenzhen | 1.0 | 0.8 | 1.1 | 1.9 |
|  | Changji | 5.7 | 8.1 | 7.2 | 9.6 |  | Qingyuan | 3.3 | 2.7 | 3.7 | 6.2 |
|  | Karamay | 1.6 | 2.3 | 2.0 | 2.7 |  | Shaoguan | 6.9 | 5.6 | 7.6 | 12.9 |
|  | Shihezi | 7.5 | 10.7 | 9.4 | 12.5 |  | Shantou | 6.6 | 5.3 | 7.3 | 12.3 |
| **NC** | **Mean** | **12.7** | **14.9** | **16.7** | **22.0** |  | Dongguan | 0.6 | 0.5 | 0.6 | 1.1 |
| **[Nationwide](file:///D:/Users/Administrator/AppData/Local/Youdao/Dict/8.6.2.0/resultui/html/index.html#/javascript:;)** | Min | 0.0 | 0.0 | 0.0 | 0.1 | Hongkong | Hongkong | 0.1 | 0.1 | 0.2 | 0.3 |
|  | Max | 76.3 | 88.2 | 99.0 | 103.5 | Macau | Macau | 0.1 | 0.1 | 0.1 | 0.2 |
|  | **Mean** | **7.2** | **8.3** | **9.2** | **12.5** | Taiwan | Taipei | 0.2 | 0.1 | 0.2 | 0.3 |
|  |  |  |  |  |  |  | Taichung | 0.2 | 0.1 | 0.2 | 0.3 |
|  |  |  |  |  |  |  | Miaoli | 1.3 | 0.9 | 1.2 | 1.9 |
|  |  |  |  |  |  |  | Hsinchu | 0.1 | 0.1 | 0.1 | 0.1 |
|  |  |  |  |  |  | Hainan | Haikou | 0.3 | 0.2 | 0.3 | 0.7 |
|  |  |  |  |  |  | **SC** | **Mean** | **1.9** | **1.7** | **2.1** | **3.9** |

**Table S10** ILCRs of PAHs in Chinese urban PM.

| Province | City | < 6 | 6-12 | 12-18 | > 18 | Province | City | < 6 | 6-12 | 12-18 | > 18 |
| --- | --- | --- | --- | --- | --- | --- | --- | --- | --- | --- | --- |
| Heilongjiang | Harbin | 3.0E-09 | 8.9E-09 | 1.5E-08 | 1.0E-07 | Yunnan | Kunming | 6.3E-08 | 9.0E-08 | 1.1E-07 | 5.6E-07 |
|  | Jiamusi | 1.2E-08 | 3.5E-08 | 5.9E-08 | 4.1E-07 |  | Yuxi | 2.4E-08 | 3.5E-08 | 4.3E-08 | 2.2E-07 |
| Jilin | Changchun | 4.6E-08 | 7.7E-08 | 1.0E-07 | 2.8E-07 | Chongqing | Chongqing | 3.5E-07 | 5.7E-07 | 7.7E-07 | 2.2E-06 |
|  | Jilin | 1.6E-06 | 2.7E-06 | 3.7E-06 | 1.0E-05 | Sichuan | Chengdu | 2.9E-08 | 5.0E-08 | 6.7E-08 | 3.0E-07 |
|  | Siping | 5.1E-07 | 8.6E-07 | 1.1E-06 | 3.2E-06 |  | Mianyang | 3.3E-08 | 5.6E-08 | 7.5E-08 | 3.4E-07 |
|  | Tonghua | 8.7E-07 | 1.5E-06 | 2.0E-06 | 5.4E-06 | Tibet | Lhasa | 2.9E-08 | 5.2E-08 | 6.2E-08 | 2.9E-07 |
|  | Baicheng | 4.7E-07 | 7.9E-07 | 1.1E-06 | 2.9E-06 | Guizhou | Guiyang | 4.4E-08 | 8.0E-08 | 1.1E-07 | 4.4E-07 |
| Liaoning | Shenyang | 2.3E-07 | 5.4E-07 | 7.5E-07 | 3.4E-06 | Anhui | Hefei | 1.2E-07 | 2.2E-07 | 2.8E-07 | 9.0E-07 |
|  | Dalian | 7.4E-08 | 1.7E-07 | 2.4E-07 | 1.1E-06 |  | Huainan | 5.1E-08 | 9.2E-08 | 1.2E-07 | 3.9E-07 |
|  | Jinzhou | 1.4E-07 | 3.2E-07 | 4.4E-07 | 2.0E-06 |  | Tongling | 1.4E-07 | 2.4E-07 | 3.2E-07 | 1.0E-06 |
|  | Fushun | 4.1E-07 | 9.5E-07 | 1.3E-06 | 6.0E-06 | Hubei | Wuhan | 4.4E-08 | 8.5E-08 | 1.1E-07 | 3.9E-07 |
|  | Anshan | 5.5E-07 | 1.3E-06 | 1.8E-06 | 8.0E-06 |  | Yichang | 4.1E-08 | 7.9E-08 | 1.0E-07 | 3.6E-07 |
|  | Panjin | 9.3E-08 | 2.1E-07 | 3.0E-07 | 1.4E-06 |  | Huangshi | 1.9E-08 | 3.6E-08 | 4.7E-08 | 1.7E-07 |
| Beijing | Beijing | 1.1E-07 | 1.9E-07 | 2.8E-07 | 1.3E-06 | Hunan | Changsha | 6.0E-08 | 8.6E-08 | 1.1E-07 | 3.4E-07 |
| Hebei | Shijiazhuang | 9.5E-08 | 1.6E-07 | 2.2E-07 | 7.8E-07 |  | Xiangtan | 5.3E-08 | 7.5E-08 | 9.3E-08 | 3.0E-07 |
|  | Zhangjiakou | 7.6E-08 | 1.3E-07 | 1.8E-07 | 6.3E-07 | Jiangsu | Nanjing | 4.9E-08 | 7.4E-08 | 9.4E-08 | 3.3E-07 |
|  | Baoding | 9.1E-08 | 1.6E-07 | 2.1E-07 | 7.5E-07 |  | Changzhou | 5.0E-08 | 7.5E-08 | 9.6E-08 | 3.3E-07 |
|  | Tangshan | 8.1E-08 | 1.4E-07 | 1.9E-07 | 6.7E-07 |  | Xuzhou | 2.8E-07 | 4.2E-07 | 5.3E-07 | 1.9E-06 |
|  | Handan | 4.5E-08 | 7.7E-08 | 1.0E-07 | 3.7E-07 |  | Wuxi | 3.0E-08 | 4.5E-08 | 5.8E-08 | 2.0E-07 |
|  | Hengshui | 2.3E-07 | 3.9E-07 | 5.3E-07 | 1.9E-06 |  | Zhenjiang | 2.1E-08 | 3.2E-08 | 4.1E-08 | 1.4E-07 |
| Henan | Zhengzhou | 3.3E-07 | 4.8E-07 | 6.2E-07 | 2.8E-06 |  | Nantong | 2.7E-08 | 4.0E-08 | 5.1E-08 | 1.8E-07 |
|  | Xinxiang | 1.2E-07 | 1.7E-07 | 2.2E-07 | 1.0E-06 | Jiangxi | Nanchang | 5.0E-08 | 1.0E-07 | 1.4E-07 | 5.1E-07 |
| Inner Mongolia | Hohhot | 4.8E-08 | 8.4E-08 | 1.2E-07 | 5.9E-07 |  | Jiujiang | 2.6E-08 | 5.2E-08 | 7.1E-08 | 2.6E-07 |
|  | Baotou | 2.7E-07 | 4.7E-07 | 6.7E-07 | 3.3E-06 | Shanghai | Shanghai | 5.8E-08 | 8.3E-08 | 1.1E-07 | 3.3E-07 |
|  | Ordos | 2.8E-08 | 5.0E-08 | 7.1E-08 | 3.5E-07 | Zhejiang | Hangzhou | 6.8E-08 | 1.0E-07 | 1.3E-07 | 4.5E-07 |
| Shandong | Jinan | 3.8E-08 | 6.8E-08 | 9.0E-08 | 4.0E-07 |  | Huzhou | 1.5E-08 | 2.2E-08 | 2.9E-08 | 1.0E-07 |
|  | Qingdao | 1.8E-08 | 3.3E-08 | 4.3E-08 | 1.9E-07 |  | Ningbo | 3.0E-08 | 4.4E-08 | 5.7E-08 | 2.0E-07 |
| Shanxi | Taiyuan | 1.1E-07 | 1.7E-07 | 2.3E-07 | 8.6E-07 |  | Wenzhou | 4.6E-08 | 6.8E-08 | 8.8E-08 | 3.1E-07 |
|  | Jinzhong | 1.1E-06 | 1.8E-06 | 2.4E-06 | 8.9E-06 |  | Taizhou | 5.3E-08 | 7.9E-08 | 1.0E-07 | 3.5E-07 |
| Tianjin | Tianjin | 7.3E-07 | 1.1E-06 | 1.5E-06 | 6.3E-06 |  | Jinhua | 2.2E-08 | 3.3E-08 | 4.3E-08 | 1.5E-07 |
| Gansu | Lanzhou | 4.7E-09 | 7.5E-09 | 1.1E-08 | 5.3E-08 | **CC** | **Mean** | **6.4E-8** | **1.0E-7** | **1.4E-7** | **4.6E-7** |
|  | Jinchang | 1.5E-07 | 2.4E-07 | 3.5E-07 | 1.7E-06 | Fujian | Xiamen | 9.3E-09 | 1.5E-08 | 2.0E-08 | 8.4E-08 |
| Shaanxi | Xi'an | 1.9E-07 | 2.8E-07 | 3.4E-07 | 1.2E-06 |  | Longyan | 9.3E-08 | 1.5E-07 | 2.0E-07 | 8.3E-07 |
|  | Yulin | 2.4E-07 | 3.5E-07 | 4.4E-07 | 1.5E-06 | Guangxi | Nanning | 8.5E-09 | 1.3E-08 | 1.7E-08 | 7.8E-08 |
| Ningxia | Yinchuan | 1.6E-07 | 2.6E-07 | 3.4E-07 | 1.0E-06 |  | Baize | 6.6E-08 | 9.9E-08 | 1.3E-07 | 6.1E-07 |
| Qinghai | Xining | 5.1E-08 | 8.1E-08 | 1.1E-07 | 2.7E-07 |  | Liuzhou | 2.2E-07 | 3.3E-07 | 4.5E-07 | 2.1E-06 |
| Xinjiang | Urumqi | 1.5E-06 | 2.9E-06 | 3.9E-06 | 1.2E-05 | Guangdong | Guangzhou | 1.8E-08 | 2.7E-08 | 3.4E-08 | 1.3E-07 |
|  | Hotan | 2.3E-07 | 4.4E-07 | 5.8E-07 | 1.8E-06 |  | Shenzhen | 3.1E-08 | 4.6E-08 | 5.8E-08 | 2.3E-07 |
|  | Changji | 1.4E-07 | 2.7E-07 | 3.6E-07 | 1.1E-06 |  | Qingyuan | 9.9E-08 | 1.5E-07 | 1.8E-07 | 7.3E-07 |
|  | Karamay | 4.0E-08 | 7.5E-08 | 1.0E-07 | 3.1E-07 |  | Shaoguan | 2.1E-07 | 3.1E-07 | 3.9E-07 | 1.5E-06 |
|  | Shihezi | 1.9E-07 | 3.5E-07 | 4.7E-07 | 1.5E-06 |  | Shantou | 2.0E-07 | 2.9E-07 | 3.7E-07 | 1.5E-06 |
| **NC** | **Mean** | **2.8E-7** | **5.0E-7** | **6.7E-7** | **2.4E-6** |  | Dongguan | 1.7E-08 | 2.6E-08 | 3.2E-08 | 1.3E-07 |
|  |  |  |  |  |  | Hongkong | Hongkong | 4.2E-09 | 6.2E-09 | 7.8E-09 | 3.1E-08 |
|  |  |  |  |  |  | Macau | Macau | 3.2E-09 | 4.8E-09 | 6.0E-09 | 2.4E-08 |
|  |  |  |  |  |  | Taiwan | Taipei | 3.3E-09 | 5.2E-09 | 7.0E-09 | 3.0E-08 |
|  |  |  |  |  |  |  | Taichung | 3.3E-09 | 5.1E-09 | 6.8E-09 | 2.9E-08 |
|  |  |  |  |  |  |  | Miaoli | 2.5E-08 | 3.9E-08 | 5.2E-08 | 2.2E-07 |
| **[Nationwide](file:///D:/Users/Administrator/AppData/Local/Youdao/Dict/8.6.2.0/resultui/html/index.html#/javascript:;)** | Min | 1.7E-9 | 2.6E-9 | 3.5E-9 | 1.5E-8 |  | Hsinchu | 1.7E-09 | 2.6E-09 | 3.5E-09 | 1.5E-08 |
|  | Max | 1.6E-6 | 2.9E-6 | 3.9E-6 | 1.2E-5 | Hainan | Haikou | 6.8E-09 | 1.0E-08 | 1.4E-08 | 8.0E-08 |
|  | **Mean** | **1.6E-7** | **2.8E-7** | **3.8E-7** | **1.4E-6** | **SC** | **Mean** | **5.6E-8** | **8.5E-8** | **1.1E-7** | **4.6E-7** |
